# Systematic revision and phylogeny of *Paragripopteryx* Enderlein, 1909 (Plecoptera: Gripopterygidae)

**DOI:** 10.1371/journal.pone.0264264

**Published:** 2022-03-03

**Authors:** Tácio Duarte, Adolfo Ricardo Calor, Pitágoras da Conceição Bispo

**Affiliations:** 1 Universidade Federal da Integração Latino Americana (UNILA), Instituto Latino-Americano de Ciências da Vida e da Natureza, Laboratório de Limnologia, Foz do Iguaçu, Paraná, Brazil; 2 Universidade Federal da Bahia (UFBA), Instituto de Biologia, Laboratório de Entomologia Aquática, Salvador, Bahia, Brazil; 3 Universidade Estadual Paulista (UNESP), Faculdade de Ciências e Letras de Assis, Laboratório de Biologia Aquática, Assis, São Paulo, Brazil; Nanjing Agricultural University, CHINA

## Abstract

Among the Neotropical gripopterygids, the genus *Paragripopteryx* occurs along the Brazilian Atlantic coast to Uruguay. Since its first recognition by Enderlein in 1909, the genus underwent a confusing taxonomic history with some combinations. In this study, we aim to revise *Paragripopteryx* and present the first morphology-based phylogeny for the genus. The analysis comprised 38 morphological characters and their respective states in 30 terminal taxa, including 13 *Paragripopteryx* species, two new species identified as belonging to *Paragripopteryx*, and 15 outgroup species, among which we can highlight 12 different South American genera and one Australian genus of Gripopterygidae. The cladistic analysis yielded a parsimonious tree for k = 3 (137 steps, consistency index = 0.445, and retention index = 0.591) where most *Paragripopteryx* are nested, except for Uruguayan *Paragripopteryx munoai*. We can then infer that in its current circumscription *Paragripopteryx* is polyphyletic. The following two species are described: *Paragripopteryx dasalmas* sp. nov. and *Paragripopteryx ogum* sp. nov. *Paragripopteryx baratinii* is designated as a *nomen dubium*. Additionally, we provide a key for species identification, updated geographical records, and illustrations for all species. As a corollary, our study gathers relevant morphological information that can help to better understand this genus and create foundations for the next steps.

## Introduction

Stoneflies (Order Plecoptera Burmeister, 1839 [1]) are relevant components of stream ecosystems all over the world, with more than 3,700 species in 17 families [2, 3]. Recent evidence suggests that the ancestors of extant stoneflies probably originated between Middle to Upper Permian period (around 265 Mya) [4], earlier than previously estimated [5, 6]. According to this view, it was during the Lower Jurassic period (around 180 Mya), in a scenario of deep changes in the Earth’s climate due to the split of the Pangaea supercontinent, that two groups, Notonemouridae and Antarctoperlaria, dispersed independently to the south, with subsequent extinction in the north [4]. Later, Antarctoperlaria diversified into Gondwana, where Gripopterygidae Enderlein, 1909 [7] diverged as an independent lineage around 139 Mya (Lower Cretaceous) [4], becoming the most diverse family in this clade, with about 320 species in 55 genera [8]. Currently, the family occurs in Australasia and South America. In South America (Andean and Neotropical regions *sensu* Morrone [9]), 28 genera are known, totaling about 110 species [10].

**Fig 1:**
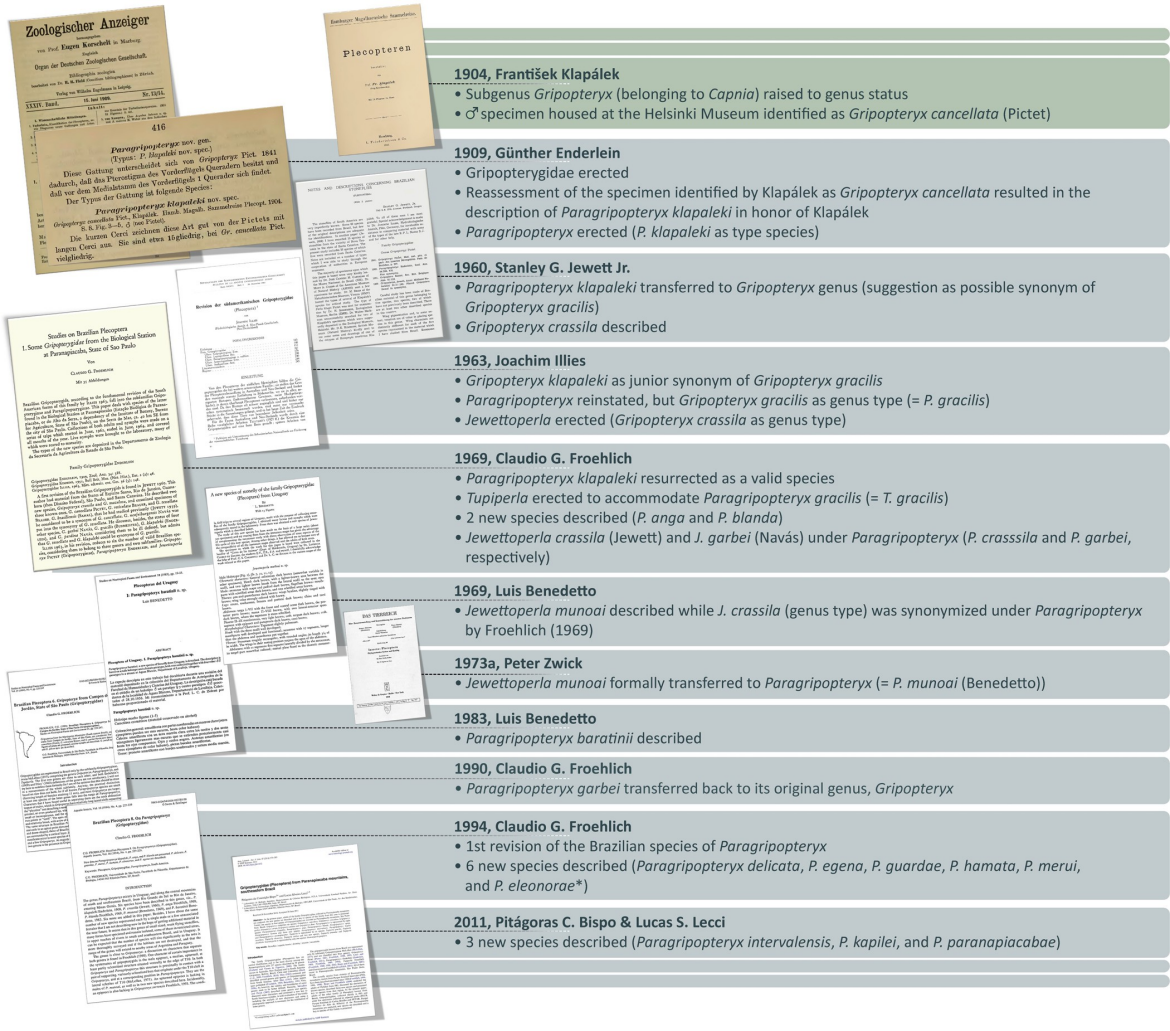
Taxonomic history of *Paragripopteryx*. *Species combined under the genus *Tupiperla* by Froehlich [19].

The genus *Paragripopteryx* Enderlein, 1909 [7] is currently represented by 14 species: 12 species in streams from the Brazilian Atlantic coast [11, 12], one of them also in streams from Province of Misiones (Argentina), and two in streams from Uruguay [13, 14] (Neotropical Region *sensu* Morrone [9]). *Paragripopteryx* was described by Enderlein [7] after the reexamination of a single male specimen housed at the Zoological Museum of Helsinki (Finland), previously identified by Klapálek [15] as *Gripopteryx cancellata* (Pictet, 1841) (Fig 1). Enderlein [7] renamed this single male as a new species in a new genus: *Paragripopteryx klapaleki* Enderlein, 1909 [7, 16]. Five decades later, in his “Notes and descriptions concerning Brazilian Stoneflies” Jewett [17] discussed the status of *P*. *klapaleki* and combined it under the genus *Gripopteryx* (Pictet, 1841) as *Gripopteryx klapaleki*. Even though he suggested that the species could be a synonym of *Gripopteryx gracilis* (Burmeister, 1839), he refrained from deciding on the issue, as the type material of *P*. *klapaleki* was lost [17, 18]. Additionally, Jewett also described *Gripopteryx crassila* Jewett, 1960 [17]. After reviewing key specimens in his “Revision der südamerikanischen Gripopterygidae (Plecoptera)”, Illies [18] reinstated the genus *Paragripopteryx* but considered *Gripopteryx klapaleki* as a junior synonym of *Gripopteryx gracilis* (= *P*. *gracilis*), in addition to erecting *Jewettoperla* to accommodate *Gripopteryx crassila* (= *Jewettoperla crassila*) and *Gripopteryx garbei* Navás, 1936 (= *Jewettoperla garbei*).

In 1969, Froehlich [16] resurrected *P*. *klapaleki* as a valid species and erected the genus *Tupiperla* Froehlich, 1969 [16] for *P*. *gracilis* (= *Tupiperla gracilis*). In this same paper, the author described two species from Brazil: *P*. *anga* Froehlich, 1969 and *P*. *blanda* Froehlich, 1969 [16]. Froehlich [16] also considered *Jewettoperla crassila* as *P*. *crassila* and *Jewettoperla garbei* as *P*. *garbei*. Subsequently, Zwick [20] formally combined the Uruguayan species *Jewettoperla munoai* Benedetto, 1969 [13] under *Paragripopteryx* (= *P*. *munoai*), and Benedetto [14] described *P*. *baratinii* Benedetto, 1983 from Uruguay. In 1990, Froehlich [21] transferred *P*. *garbei* back to its original genus, *Gripopteryx* (= *G*. *garbei*). In 1994, Froehlich [22] carried out the most comprehensive study of the genus *Paragripopteryx*, reviewing all of the Brazilian species and describing six new species: *P*. *delicata* Froehlich, 1994, *P*. *egena* Froehlich, 1994, *P*. *guardae* Froehlich, 1994, *P*. *hamata* Froehlich, 1994, *P*. *merui* Froehlich, 1994, and *P*. *eleonorae* Froehlich, 1994. In 1998 [19], *P*. *eleonorae* was transferred to *Tupiperla* by the same author. Finally, in 2011 Bispo and Lecci [12] described three new Brazilian species based on males: *P*. *intervalensis* Bispo and Lecci, 2011, *P*. *kapilei* Bispo and Lecci, 2011, and *P*. *paranapiacabae* Bispo and Lecci, 2011.

Even though the *Paragripopteryx* species reveal only a slight morphological variation, the assessment of the morphological characters may shed light on the monophyly and phylogenetic position of the genus, besides enabling the understanding of the phylogenetic relationship between the species. In this study, we study the morphology of *Paragripopteryx* using a cladistic approach to test the genus monophyly and propose a hypothesis for the phylogenetic relationship between species. In addition, we revise the genus taxonomy, describe new species, and present an identification key.

## Material and methods

### Species studied

We studied specimens housed in scientific collections and specimens we collected. Considering specimens from scientific collections, they are housed in the institutions specified in Table 1. Our specimen sampling followed Brazilian laws and were authorized by the SISBIO-ICMBio (Biodiversity Authorization and Information System, Chico Mendes Institute for Biodiversity Conservation, numbers 53570–1 and 3363–1). The dataset comprised 30 terminal taxa, including 13 *Paragripopteryx* species, two new species identified as belonging to *Paragripopteryx*, and 15 outgroup species, among which we can highlight 12 different South American genera and one Australian genus of Gripopterygidae. The outgroup selection was based on McCulloch et al. [6] and Pessacq et al. [23].

**Table 1:** Species studied for the phylogenetic proposition, the institutions where the specimens are deposited, and biogeographical occurrences. Andean Region, Transition Zone, and Neotropical Region (*sensu* Morrone [9]).

Groups	Species examined	Institutions where the material is deposited	Occurrence region
**Outgroup**	*Alfonsoperla flinti* McLellan & Zwick, 2007 [24]; *Aubertoperla illiesi* (Froehlich, 1960) [25]; *Ceratoperla fazi* (Navás, 1934) [26]; *Limnoperla jaffueli* (Navás, 1928) [27]; *Potamoperla myrmidon* (Mabille, 1891) [28]; *Rhithroperla rossi* (Froehlich, 1960) [25]; *Teutoperla rothi* Illies, 1963 [18]; *Uncicauda testacea* (Vera, 2006) [29].	CIEMEP, Argentina: additional material	Andean Region (Argentine and Chilean Patagonia)
	*Claudioperla tigrina* (Klapálek, 1904) [15].	MZSP, Brazil: additional material	Andean, Neotropical, and Transition Zone (along the Andes from Colombia to North Argentina and Central Chile)
	*Gripopteryx cancellata* (Pictet, 1841) [30]; *Guaranyperla guapiara* Froehlich, 2001 [31]; *Guaranyperla nitens* Froehlich, 2001 [31]; *Tupiperla gracilis*; *Tupiperla robusta* Froehlich, 1998 [19].	MZSP, Brazil: type series	Neotropical Region (Brazilian Atlantic coast).
	*Riekoperla karki* McLellan, 1971 [32].	CIEMEP, Argentina: additional material	Australian Region (Australian coast, Victory).
**Ingroup**	*Paragripopteryx anga*; *P*. *blanda*; *P*. *delicata*; *P*. *egena*; *P*. *guardae*; *P*. *hamata*; *P*. *intervalensis*; *P*. *kapilei*; *P*. *klapaleki*; *P*. *merui*; *P*. *paranapiacabae*.	MZSP, Brazil: type series UFV, Brazil: additional material	Neotropical Region (Brazilian Atlantic coast)
	*Paragripopteryx dasalmas* sp. nov.	MZSP, Brazil: type series UFBA, Brazil: paratypes	
	*Paragripopteryx ogum* sp. nov.	MZSP, Brazil: type series	
	*Paragripopteryx crassila*.	CAS, The United States of America: type series	
	*Paragripopteryx munoai*.	UDELAR, Uruguay: type series	Neotropical Region (Uruguayan Atlantic coast)

Depositories’ name in the subsection “Abbreviations”.

*Paragripopteryx crassila* was analyzed by taking into account the original description provided by Jewett [17] and the photos of the holotype sent to us by Dr. Christopher Grinter from the California Academy of Science (CAS), United States of America. Specimens of *P*. *baratinii* were not available for study. Although the material regarding this species was deposited by Benedetto [14] at the UDELAR, Montevideo, Uruguay, the type material was not found in such institution (Dr. Enrique Morelli, [Unpublished]). According to Benedetto [14], two male paratypes of *P*. *baratinii* were also sent to the collection of the Limnological Station of the Max-Planck Institute, Schlitz, Germany. However, Dr. Peter Zwick [Unpublished], former director of the station, reported that the material of *P*. *baratinii* had never been at the Max-Planck Institute collection. Therefore, *P*. *baratinii* type series is currently considered lost and the species doubtful. The original description of this species is incomplete and useless for the phylogenetic analyses, justifying why *P*. *baratinii* was not included in our study.

The following original papers were relevant to complement the morphological characters and/or propose the primary homologies: Jewett [17], Illies [18], Benedetto [13], Froehlich [16, 19, 21, 22, 31, 33], and Bispo and Lecci [12]. The nomenclature for wing venation followed Béthoux [34], while for genitalia structures it was based on the work by Froehlich [16, 22].

### Microscopy and image processing

The specimens studied were examined and photographed by the authors through a Leica M205A stereomicroscope. The photographs were processed using the image editing software Adobe Photoshop CC®. The line drawings were prepared with the aid of a camera lucida and vectored on Adobe Illustrator CC®. The species registration map was prepared using QGis software v. 3.10 [35] and the online public domain platform SimpleMappr [36]. For the map, we used the collection sites specified in the literature, the data from material deposited in museums and the data from the new specimens sampled by us.

### Morphological treatments

For the 30 terminal taxa, we selected 38 morphological characters (27 binary and 11 multistate) and their respective states. The phylogenetic characters were proposed following Sereno [37]. The character matrix was built with the aid of Mesquite software v. 3.61 [38] and exported to the format employed in the Tree analysis using New Technology software (TNT v. 1.5) [39]. The morphological characters were used for both nymphs and adults, except for species with unknown nymphs. The missing and inapplicable data were coded as “?” and “-”, respectively, whereas the ambiguous character state was marked with “*”.

### Cladistic analysis

The parsimony analyses were run considering all characters as nonadditive [40]. The dataset was analyzed using the “Traditional search” command in TNT software, with Tree Bisection Reconnection (TBR) branch swapping, 10,000 replications, 100 trees to save per replication, and collapsing trees after the search.

The analyses were implemented under Implied Weighting (IW) against homoplastic characters [41, 42]. According to Prendini [43], under different weighting schemes it is possible to recover more reliable relationships. Thus, we employed the scheme of concavity index variation (k = 3–15) to better understand the topological impacts of optimization and character changes in the most parsimonious trees. Furthermore, the value of k was also estimated using the *setk*.*run* script (S. Arias [Unpublished]). The relative Bremer support [44] was used to estimate the branch support, with 1000 suboptimal trees up to five steps longer (obtained from traditional search).

### Nomenclatural acts

The electronic edition of this article conforms to the requirements of the amended International Code of Zoological Nomenclature. Therefore, the new names contained herein are available under this Code in the electronic edition of this article. This published work and the nomenclatural acts it contains have been registered in ZooBank, the online registration system for the ICZN. The ZooBank LSIDs (Life Science Identifiers) can be resolved and the associated information viewed through any standard web browser by appending the LSID to the prefix “http://zoobank.org/”. The LSID for this publication is: *urn*:*lsid*:*zoobank*.*org*:*pub*:*7D9434BA-2533-4DDF-B1B8-D21081FE53CB*. The electronic edition of this work was published in a journal with an ISSN, and has been archived and is available from the following digital repositories: LOCKSS, Periódicos CAPES (https://www.periodicos.capes.gov.br).

### Abbreviations

#### Morphology

AA1 –First anterior anal vein; AA2 –Second anterior anal vein; Ant–Antennae; Brls–Black rod-like setae; Cc–Cercus; CuA–Anterior cubitus vein; CuP–Posterior cubitus vein; Dt–Denticles; Ep–Epiproct; Fe–Femur; Hd–Head; M–Media vein; Ma–Membranous area; PC–Pterostigmatic cell; Pnt–Pronotum; Pp–Paraprocts; Ptc–Pterostigmatic crossvein; RA–Anterior radius vein; RP–Posterior radius vein; RPc–RP crossvein; Sat–Subapical tooth (paraprocts); Sc–Subcosta vein; Sp–Subgenital plate; T8 –Tergum 8; T9 –Tergum 9; T10 –Tergum 10; T10E –Tergum 10 extension; Tb–Tibia; Ts–Tarsi; Tth–Tooth.

#### Depositories

CAS–California Academy of Science, San Francisco, California State, United States of America; CIEMEP–Esquel de Montaña and Estepa Patagónica Research Center, Esquel, Chubut Province, Argentina; MZSP–Museum of Zoology, University of São Paulo, São Paulo state, Brazil; UDELAR–University of the Republic, Montevideo, Montevideo Department, Uruguay; UFBA–Natural History Museum of Bahia, Federal University of Bahia, Salvador, Bahia state, Brazil; UFV–Museum of Entomology, Federal University of Viçosa, Viçosa, Minas Gerais state, Brazil.

## Results

### Morphological characters

We studied the morphology of *Paragripopteryx* and some other Gripopterygidae species. As a result, we selected and encoded 12 characters for the nymphs and 26 for the adults, reaching 38 characters (S1 Table). Below is the list of characters.

Nymphs–Thorax (characters 1–7)

1. Thorax; type of bristles: (0) only setiform bristles; (1) vesicular bristles; (2) claviform bristles (homoplasy value (H) = 0.25000; consistency index (CI) = 0.667; retention index (RI) = 0.909). Froehlich [16, 31] described the claviform and vesicular bristles in nymphs of Brazilian Gripopterygidae.
2. Pronotum; cuticular surface structure: (0) without small cuticular projections; (1) with small cuticular projections (like spines or elevations) (H = 0.25000; CI = 0.500; RI = 0.500). Illies [18] provided some figures of the South American genera of Gripopterygidae in which this character can be observed.
3. Pronotum; paranota: (0) absent; (1) present (H = 0.00000; CI = 1.000; RI = 1.000). Froehlich [31] described the paranota in nymphs of *Guaranyperla*.
4. Metanotum; mid-distal margin (between wingpads), shape: (0) circular, concave; (1) circular, convex; (2) angular excised (pyramid-shaped); (3) angular (V-shaped); (4) straight; (5) bilobated (W-shaped) (H = 0.40000; CI = 0.714; RI = 0.667).
5. Femur; extensor margin, fringe of bristles: (0) absent; (1) present (H = 0.50000; CI = 0.250; RI = 0.250).
6. Tibia; extensor margin, fringe of bristles: (0) absent; (1) present (H = 0.50000; CI = 0.250; RI = 0.571).
7. Tarsi; ventral face of 3rd tarsal segment, type of bristles: (0) spinose/spine-like setae; (1) setiform, small robust setae; (2) setiform, small thin setae (H = 0.25000; CI = 0.667; RI = 0.750). McLellan and Zwick [24] described differences among the types of bristles in the tarsal segment.
  Nymphs–Abdomen (characters 8–12)
8. Abdomen; distal margin of terga 1–9, ring of bristles: (0) absent; (1) present (H = 0.00000; CI = 1.000; RI = 1.000).
9. Abdomen; distal margin of terga 1–9, type of bristles: (0) setiform; (1) vesicular; (2) claviform (H = 0.00000; CI = 1.000; RI = 1.000). Character applicable to taxa showing ring of bristles on the distal margin of terga 1–9 (character 8(1)).
10. Abdomen; terga 1–9, row of mid-dorsal spines: (0) absent; (1) present. (H = 0.25000; CI = 0.500; RI = 0.000).
11. Abdomen; T10, distal margin, shape: (0) curved; (1) trapezoid; (2) triangular; (3) spine-shaped (H = 0.57143; CI = 0.429; RI = 0.500).
12. Paraprocts; apex, general shape: (0) rounded; (1) square; (2) triangular (H = 0.66667; CI = 0.250; RI = 0.250).
  Adults–Thorax (characters 13–21)
13. Forewings; pterostigmatic crossveins: (0) absent; (1) present (H = 0.25000; CI = 0.500; RI = 0.923).
14. Forewings; RP vein, shape: (0) unforked; (1) forked (H = 0.25000; CI = 0.500; RI = 0.500).
15. Forewings; RP forked, crossveins: (0) absent; (1) present (H = 0.25000; CI = 0.500; RI = 0.667). Character applicable to taxa showing forked RP vein (character 14(1)).
16. Forewings; CuA vein, shape: (0) unforked; (1) forked (H = 0.25000; CI = 0.500; RI = 0.000).
17. Hind wings; inferior branch of M vein in relation to CuA vein, shape: (0) partially fused, separating near the wing margin; (1) completely fused (H = 0.40000; CI = 0.333; RI = 0.000).
18. Hind wings; 6th anal vein, shape: (0) 6th anal vein free of wing margin; (1) 6th anal vein fused to the wing margin (H = 0.25000; CI = 0.500; RI = 0.500).
19. Femur; flexor margin, distal spine (disto-ventral spine): (0) absent; (1) present (H = 0.00000; CI = 1.000; RI = 1.000).
20. Tibia; distal region, spurs: (0) absent; (1) present (H = 0.00000; CI = 1.000; RI = 1.000).
21. Tarsi; basal tarsal segment in relation to apical segment: (0) shorter than apical segment, half the length; (1) subequal or equal to the apical segment (H = 0.00000; CI = 1.000; RI = 1.000).
  Adults–Abdomen (characters 22–38)
22. Abdomen; T10, lateral clefts separating lateral sclerites from the anterior sclerite: (0) absent; (1) present (H = 0.62500; CI = 0.167; RI = 0.000).
23. Abdomen; T10, anterior sclerites, fusion (inner margin): (0) completely fused; (1) contacting in a small point or bridge; (2) separated by a membrane (H = 0.50000; CI = 0.400; RI = 0.500).
24. Abdomen; T10, anterior sclerites in relation to central sclerite: (0) completely fused to central sclerite; (1) separated from central sclerite by a membrane; (2) fused to central sclerite but distinguishable from it by a suture (H = 0.25000; CI = 0.667; RI = 0.667).
25. Abdomen; T10, central sclerite, distal margin, condition: (0) not protruding; (1) protruding (H = 0.57143; CI = 0.200; RI = 0.000).
26. Abdomen; T10, central sclerite (extension), distal margin, shape: (0) not bilobated; (1) bilobated (H = 0.62500; CI = 0.167; RI = 0.375). Character applicable to taxa showing protruding distal margin of central sclerite (character 25(1)).
27. Abdomen; T10, central sclerite, tooth at the distal margin: (0) absent; (1) present (H = 0.25000; CI = 0.500; RI = 0.800).
28. Abdomen; T10, central sclerite, number of teeth at the distal margin: (0) one; (1) two (H = 0.00000; CI = 1.000; RI = 1.000). Character applicable to taxa showing tooth on the distal margin of central sclerite (character 27(1)).
29. Abdomen; T10, posterior sclerite: (0) absent; (1) present (H = 0.00000; CI = 1.000; RI = 1.000).
30. Epiproct sclerotized: (0) absent; (1) present (H = 0.57143; CI = 0.200; RI = 0.500).
31. Epiproct; general shape: (0) hook-shaped (tip facing outwards); (1) hook-shaped (tip facing inwards); (2) falciform; (3) shortened finger-shaped; (4) elongated finger-shaped; (5) spoon-shaped (H = 0.40000; CI = 0.714; RI = 0.778). Character applicable to taxa showing epiproct (character 30(1)).
32. Epiproct; row of dorsal denticles (dorsal teeth): (0) absent; (1) present (H = 0.40000; CI = 0.333; RI = 0.750). Character applicable to taxa showing epiproct (character 30(1)).
33. Paraprocts; distal third, narrow (constrict): (0) absent; (1) present (H = 0.50000; CI = 0.250; RI = 0.571).
34. Paraprocts; distal third, black rod-like setae/bristles (stout setae): (0) absent; (1) present (H = 0.00000; CI = 1.000; RI = 1.000).
35. Paraprocts; distal margin, shape: (0) rounded; (1) almost pointed (H = 0.62500; CI = 0.167; RI = 0.167).
36. Subgenital plate (male); distal margin, shape: (0) general triangular with rounded tip; (1) trapezoid with slightly straight tip; (2) trapezoid with notched distal margin; (3) curved; (4) straight (H = 0.50000; CI = 0.571; RI = 0.700).
37. Subgenital plate (female); middle region, unsclerotized spot: (0) absent; (1) present (H = 0.57143; CI = 0.200; RI = 0.200).
38. Subgenital plate (female); shape: (0) ovoid with cut/straight distal margin; (1) ovoid with rounded distal margin; (2) trapezoid with notched distal margin; (3) trapezoid with rounded distal margin; (4) square with notched distal margin; (5) square with slightly curved distal margin (H = 0.62500; CI = 0.500; RI = 0.444).

### Morphological phylogeny

**Fig 2:**
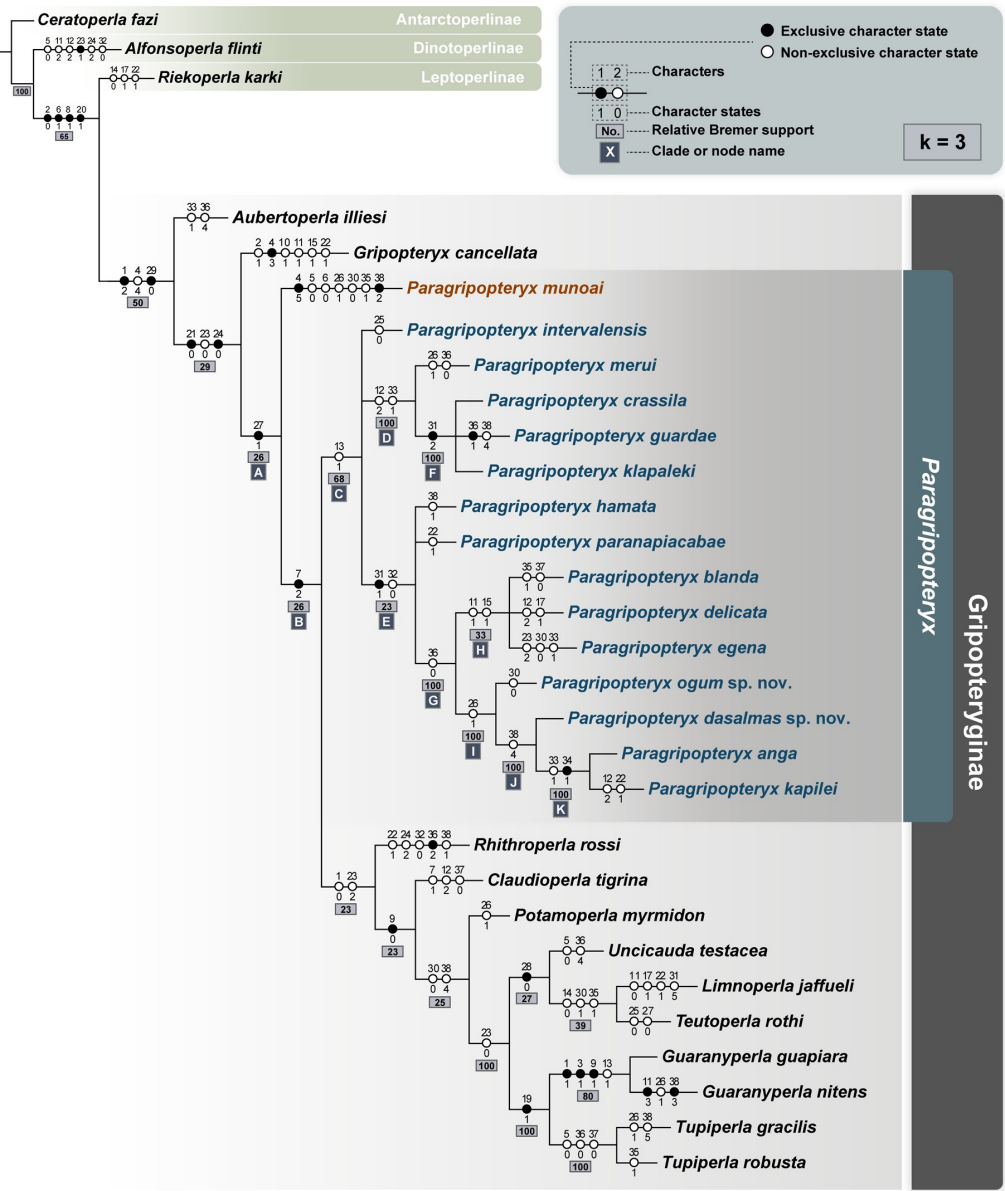
Phylogenetic hypotheses of *Paragripopteryx*. Analysis under IW scheme (k = 3); most *Paragripopteryx* (written in blue) are nested in a clade, except for *P*. *munoai* (written in red).

The parsimony analyses under IW yielded two different trees (Length (L) = 137, consistency index (CI) = 0.445, and retention index (RI) = 0.591). We recovered a single tree for k = 3–5 and the same tree when using the script *setk*.*run* (k = 3.75), where most *Paragripopteryx* are nested (Clade C), except for *P*. *munoai* (Fig 2). In contrast, for k = 7–15 we found a strict consensus tree for two topologies in each value of k (7, 9, 11, 13, 15), where most *Paragripopteryx* are nested in a poorly resolved clade, except for *P*. *munoai* (S1 Fig). We selected a strong concavity for IW schemes (k = 3) to decrease the weight of homoplasies and better resolve conflicts between the distribution of homoplastic characters [41]. Therefore, the results presented hereinafter are based on the value of k = 3.

Our results indicate that *Paragripopteryx* is polyphyletic, with *P*. *munoai* nested outside Clade C, which encompasses other species of the genus (Fig 2). Instead, *P*. *munoai* is nested at the base of a large clade composed of other species of *Paragripopteryx* + Gripopteryginae (except for *Aubertoperla illiesi* (Froehlich, 1960) [25] and *Gripopteryx cancellata*) (Clade A; Fig 2). This large clade (Clade A, which also includes *P*. *munoai*) is supported by a single synapomorphy in all values of k: the presence of teeth on the distal margin of the central sclerite (character 27(1)).

After the divergence of *P*. *munoai*, the Clade B emerges, which encompasses other species of *Paragripopteryx* and most species of Gripopteryginae included in our study. A single synapomorphy supports Clade B: the ventral face of the third tarsal segment in nymphs provided with small thin setae (character 7(2)). Inside Clade B emerges Clade C, which is supported by only one synapomorphy (the presence of pterostigmatic crossveins in the forewings, character 13(1)) and encompasses all species of *Paragripopteryx*, except for *P*. *munoai* (Fig 2).

Inside Clade C, *Paragripopteryx* species are nested in a polytomy composed of *P*. *intervalensis* plus Clades D and E (Fig 2). Clade D splits into *P*. *merui* as sister of Clade F (*P*. *crassila*, *P*. *guardae*, and *P*. *klapaleki*), being supported by two synapomorphies: the general triangular shape of the apex of the paraprocts in nymphs (character 12(2))–a non-exclusive character state shared with *P*. *delicata* and *P*. *kapilei* and convergent with *Claudioperla tigrina* (Klapálek, 1904) [15]; and the narrow (constricted) shape of the distal third of the paraprocts in male adults (character 33(1))–a character state shared with *P*. *egena* and Clade K. Clade F (Fig 2), in turn, is supported by a single synapomorphy: the falciform epiproct (character 31(2)).

Clade E (Fig 2) includes all other species of *Paragripopteryx* and is supported by two synapomorphies: the hook-shaped epiproct (tip facing inwards) (character 31(1)) and the absence of a row of dorsal denticles in the epiproct (character 32(0))–a non-exclusive character state convergent with *Rhithroperla rossi* (Froehlich, 1960) [25]) (Fig 2). Inside Clade E, *P*. *hamata* and *P*. *paranapiacabae* share a polytomy with Clade G (Fig 2), with the latter being supported by a single synapomorphy: the distal margin of the subgenital plate (male) with rounded tip (character 36(0))–a character state shared with *P*. *merui* and convergent with *Tupiperla* species.

Clade G splits into Clade H (*P*. *blanda*, *P*. *delicata*, and *P*. *egena*) and Clade I (*P*. *ogum* sp. nov., *P*. *dasalmas* sp. nov., *P*. *anga*, and *P*. *kapilei*) (Fig 2). Two synapomorphies support Clade H: the nymphs with distal margin of T10 trapezoidal (character 11(1)) and the presence of crossvein in the forewing RP fork (character 15(1)), both characters convergent with *Gripopteryx cancellata*. Clade I, on the other hand, is supported by a single synapomorphy: the central sclerite (extension) in T10 with bilobated distal margin (character 26(1))–a non-exclusive character state shared with *P*. *merui* and *P*. *munoai* and convergent with *Potamoperla myrmidon* (Mabille, 1891) [28], *Guaranyperla nitens* Froehlich, 2001 [31], and *Tupiperla gracilis*. Inside Clade I emerges Clade J (*P*. *dasalmas* sp. nov. plus Clade K) (Fig 2), which is supported by a single synapomorphy: the subgenital plate (female) with notched distal margin (character 38(4))–a non-exclusive character state shared with *P*. *guardae* and convergent with *Potamoperla myrmidon*, *Uncicauda testacea* (Vera, 2006) [29], *Limnoperla jaffueli* (Navás, 1928) [27], *Teutoperla rothi* Illies, 1963 [18], *Guaranyperla guapiara* Froehlich, 2001 [31], and *Tupiperla robusta* Froehlich, 1998 [19]). Lastly, Clade K (*P*. *anga* and *P*. *kapilei*) is supported by two synapomorphies (Fig 2): the narrow (constrict) shape of the distal third of the paraprocts in males (character 33(1))–a character state shared with *P*. *egena* and Clade D and convergent with *Aubertoperla illiesi*; and the presence of black rod-like setae/bristles (stout setae) in the distal region of the paraprocts (character 34(1)).

### Taxonomic treatment

Phylogenetic analyses revealed that in its current circumscription *Paragripopteryx* is not monophyletic, as *P*. *munoai* is not nested in the same clade of other species of the genus. However, although our data suggest the need to describe a new genus to allocate *P*. *munoai*, we took a more conservative decision. The description of a new genus would be better supported after collecting new specimens of *P*. *munoai*, specimens in the type locality of *P*. *baratinii*, and the proposition of a phylogeny based on total evidence (morphological + molecular data). In this study, we revised all species of the genus, including *P*. *munoai* and *P*. *baratinii* (through the literature), and made taxonomic adjustments, which resulted in the consideration of *P*. *crassila* as a junior synonym of *P*. *klapaleki* and in the designation of *P*. *baratinii* as a *nomen dubium*. Additionally, we described two new species (*P*. *dasalmas* sp. nov. and *P*. *ogum* sp. nov.) along with the nymphs of other two species (*P*. *intervalensis* and *P*. *kapilei*), updated the distribution of each species, and presented an identification key for adults.

### Genus *Paragripopteryx* Enderlein, 1909

*Paragripopteryx* Enderlein, 1909 [7]—Zoologischer Anzeiger, 34: 416 (type of the genus: *Paragripopteryx klapaleki* Enderlein, 1909 [7] = *Gripopteryx cancellata* Klapálek, 1904 [15] (nec Pictet, 1841 [30]), by original designation and monotype).

*Gripopteryx* Jewett, 1960 [17]—Arquivos do Museu Nacional (Rio de Janeiro), 50: 167–180 (synonymy (partim)).

*Paragripopteryx* Illies, 1963 [18]—Mitteilungen der Schweizerischen Entomologischen Gesellschaft, 36: 178 (revalidated).

*Jewettoperla* Illies, 1963 [18]—Mitteilungen der Schweizerischen Entomologischen Gesellschaft, 36: 184 (type of the genus: *Gripopteryx crassila* Jewett, 1960 [17], by original designation. Synon. nov.).

*Paragripopteryx* Froehlich, 1969 [16]—Studies on Neotropical Fauna and Environment, 6(1): 17–39 (in Illies, 1966 [45] after the manuscript in detail under Froehlich, 1964 [46] included in the catalog).

*Paragripopteryx* Zwick, 1973 [20]—Das Tierreich, 94: 210 (catalog).

*Paragripopteryx* Froehlich, 1994 [22]—Aquatic Insects, 16(4): 227–239 (review and new species).

*Paragripopteryx* Stark, Froehlich and Zúñiga, 2009 [47]—Aquatic biodiversity in Latin America, 91 (book review).

*Paragripopteryx* Froehlich, 2010 [11]—Illiesia, 6(12): 135 (catalog).

*Paragripopteryx* Bispo and Lecci, 2011 [12]—Annales de Limnologie–International Journal of Limnology, 47: 376–379 (new species).

Type species: *Paragripopteryx klapaleki* Enderlein, 1909.

#### Diagnosis

*Nymph*: body compact, covered with spaced claviform bristles; pronotum almost square, narrower than head (subequal in *P*. *munoai*); metanotum with mid-posterior margin straight (bilobated in *P*. *munoai*). Legs short; femora and tibiae extensor margin with a fringe of bristles, sometimes weak (absent in *P*. *munoai*), without femoral spine on the flexor margin of the femur, tibial spurs present. No abdominal or thoracic spines on terga. *Adult*: wings elongated (half-elliptical in *P*. *munoai*); forewings may have weak dark spots bordering veins and crossveins, not scattered, RA unforked, RP short and forked, CuA long and forked, pterostigmatic crossveins present (absent in *P*. *munoai*); hind wings with inferior branch of M3+4 and CuA veins short, forked close to the margin of the wing. Male T10E ends in two teeth, extension sometimes not distinctly protruded; latero-dorsal clefts small or inconspicuous; posterior sclerite absent. Epiproct upturned, sclerotized, laterally flattened and directed forward, inner margin may have denticles (epiproct sclerotized absent in *P*. *egena*, *P*. *munoai*, and *P*. *ogum* sp. nov.). Female subgenital plate square, possibility of notched end. Eggs flattened and ellipsoid with a cortical layer [16, 21, 22].

#### Comments

Based on phylogenetic inference, most *Paragripopteryx* species are nested by the presence of pterostigmatic crossveins in the forewings, except for *P*. *munoai*. Among the Neotropical genera, adults of *Paragripopteryx* can be partly confused with those of some *Gripopteryx* species. However, a combination of characters can easily distinguish between them. Nymphs of *Paragripopteryx* differ from *Gripopteryx* by lack of thoracic and abdominal dorsal spines. Male adults of *Paragripopteryx* present a T10E that ends in two teeth (absent in *Gripopteryx*), and the adults are smaller than those of most *Gripopteryx* species [12, 21, 22].

#### Geographic distribution

**Fig 3:**
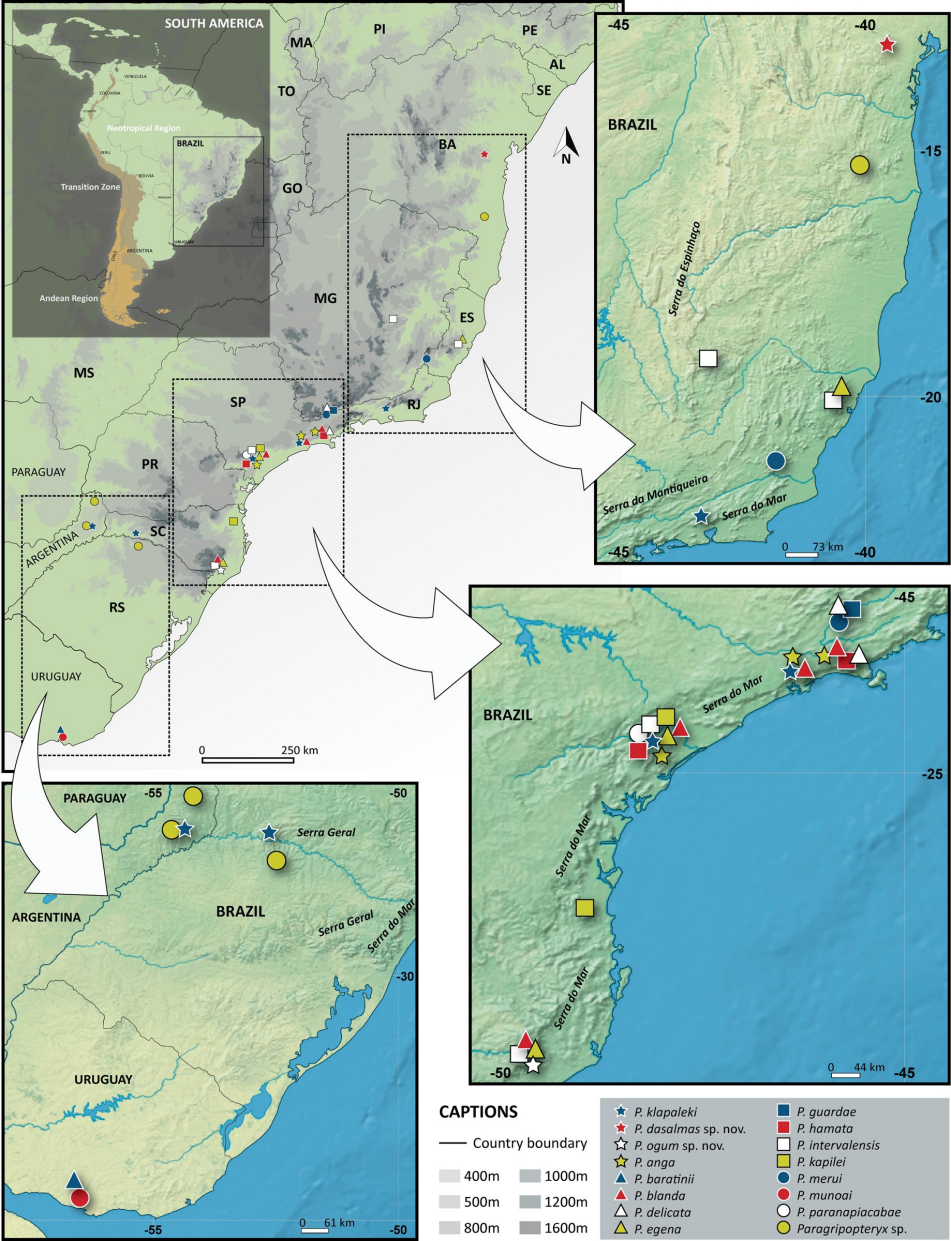
Distribution map. Map of South America highlighting the Neotropical Region (in light green) with records of *Paragripopteryx* along the Brazilian coastal mountains, from northeastern Bahia to southeastern and southern Brazil, reaching the Province of Misiones (Argentina), where some specimens were recorded, and the Uruguayan coast, where *P*. *baratinii* and *P*. *munoai* were sampled. (Reprinted from https://www.simplemappr.net under a CC BY license, with permission from David Shorthouse, original copyright 2021.).

*Paragripopteryx* occurs mainly along the Brazilian coastal mountains, where there are currently 13 species (all of them belonging to Clade C) recorded from Bahia state to Santa Catarina state, Brazil (Fig 3); specimens of the genus were also reported in Rio Grande do Sul state, Brazil [48] and in Province of Misiones, Argentina [49]. In addition, two species (*P*. *baratinii* and *P*. *munoai*) were recorded in the Uruguayan coast [13, 14] (Fig 3).

### *Paragripopteryx klapaleki* Enderlein, 1909, comb. nov

(Fig 4A–4F)

**Fig 4:**
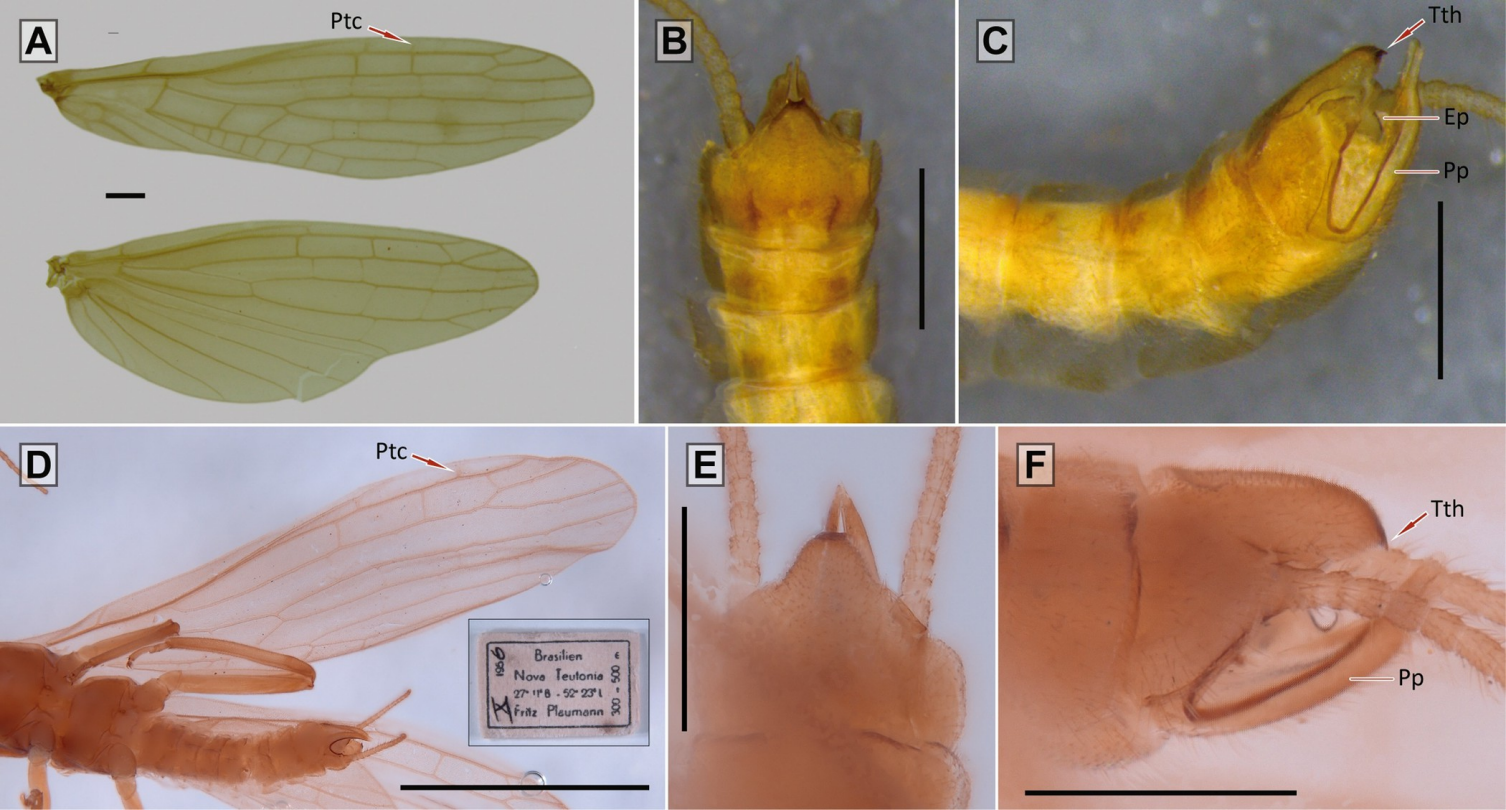
Morphological characters of *Paragripopteryx klapaleki*. *Paragripopteryx klapaleki*: **(A)** adult male, fore- and hind wings. **(B**, **C)** Male terminalia in dorsal and lateral views, respectively. *Paragripopteryx crassila*: **(D)** holotype adult male, detail of forewing. **(E**, **F)** Male terminalia in dorsal and lateral views, respectively (Scale bars: A, B, C, E, F: 0.5 mm; D: 2.0 mm).

*Paragripopteryx klapaleki* Enderlein, 1909 [7]—Zoologischer Anzeiger, 34: 416 (= *Gripopteryx cancellata* Klapálek, 1904 [15] (nec Pictet, 1841 [30])—Ergebnisse der Hamburger Magalhaensischen Sammelreise 1892/93, 7(5): 8–9, Figs 3–5, Petrópolis, R.J., Brazil; probably lost, cf. Jewett, 1960: 170 [17]).

*Gripopteryx crassila* Jewett, 1960 [17]—Arquivos do Museu Nacional (Rio de Janeiro), 169 (male, female, Fig 1, 1A and 1B).

*Gripopteryx klapaleki* Jewett, 1960 [17]—Arquivos do Museu Nacional (Rio de Janeiro), 170 (notes).

*Paragripopteryx gracilis* Illies, 1963 [18]—Mitteilungen der Schweizerischen Entomologischen Gesellschaft, 32: 179 (synonym).

*Jewettoperla crassila* Illies, 1963 [18]—Mitteilungen der Schweizerischen Entomologischen Gesellschaft, 36: 185 (comb. nov.).

*Paragripopteryx crassila* Illies, 1966 [45]—Das Tierreich, 82: 44 (catalog).

*Paragripopteryx klapaleki* Illies, 1966 [45]—Das Tierreich 82: 46 (catalog).

*Paragripopteryx crassila* Froehlich, 1969 [16]—Studies on Neotropical Fauna and Environment, 6(1): 19 (comb. nov.).

*Paragripopteryx klapaleki* Froehlich, 1969 [16]—Studies on Neotropical Fauna and Environment, 6(1): 19 (revalidation, males, females, nymphs, Figs 1–10).

*Paragripopteryx klapaleki* Zwick, 1973 [50]—Annales Zoologici, 31(16): 476 (comments).

*Paragripopteryx klapaleki* Froehlich, 1994 [22]—Aquatic Insects, 16(4): 228 (review).

*Paragripopteryx crassila* Froehlich, 2010 [11]—Illiesia, 6(12): 135 (catalog).

*Paragripopteryx klapaleki* Froehlich, 2010 [11]—Illiesia, 6(12): 136 (catalog).

*Paragripopteryx klapaleki* Bispo and Lecci, 2011 [12]—Annales de Limnologie–International Journal of Limnology, 47: 376 (remarks).

*Paragripopteryx klapaleki* Gonçalves, Novaes and Salles, 2017 [51]—Zootaxa, 4291(3): 570 (new record; see *P*. *merui*).

*Material examined*. BRAZIL: São Paulo, Estação Biológica de Paranapiacaba, 6.viii.1963, 1 female (at light) (MZSP); same data, except: 27.viii.1963, 3 females; same data, except: 5.xi.1963, 2 females; same data, except: 27.xi.1963, 2 males, 2 females; same data, except: 13.xii.1963, 3 females (2 at light); same data, except: 27.iii.1964, 2 males, 1 female; same data, except: 23.iv.1964, 1 male; same data, except: 20.v.1964, 1 female.—Adults emerged in the laboratory: viii.1962, 1 male; ix.1962, 1 female; iv.1963, 1 male; vii.1963, 3 males, 5 females; viii.1963, 3 females; ix.1963, 3 females; x.1963, 1 male, female; vi.1964, 2 males, 1 female.—Cast exuviae (last nymphal instar): 19.vii.1963, 2 females; 15.x.1963, 1 male, 2 females; 23.iv.1964, 1 female; 20.v.1964, 1 male, 1 female.—***Other material***: BRAZIL: Santa Catarina, Nova Teutônia, 1 male, x.1962 (*Fritz Plaumann*) (material deposited at the MZSP as *P*. *klapaleki*—MZUSP000359) (*new record*); BRAZIL: Santa Catarina, Nova Teutônia: 1 male, 1 female, x.1956 (*Fritz Plaumann*) (both holotype and allotype deposited in the collection of CAS as *Paragripopteryx crassila*).

#### Diagnosis

*Nymph*: body hairs mostly short, claviform (Fig 8 in [16]). Dorsal row of long bristles of femora and tibiae not thick [16]. *Adult*: generally brown to dark brown. Male forewings with pterostigmatic crossveins (in some males they may be weak) (Fig 4A and 4D). Male T10E forms a small process ending in two teeth close to each other (Fig 4B and 4E). Male paraprocts elongate, narrow and almost straight, apical region narrower, end rounded (Fig 4C and 4F). Male epiproct bends upward, almost falciform (Fig 4C). Female subgenital plate with a sclerotized band on each side and a central area not sclerotized (Fig 4 in [16]). Female paraprocts pointed.

#### Comments

In his “Studies on Brazilian Plecoptera 1”, Froehlich [16] presented comments about the morphology of *P*. *klapaleki* and considered *Jewettoperla* as a synonym of *Paragripopteryx*. Thus, Froehlich [16] considered *Jewettoperla crassila* as *P*. *crassila*. The paraprocts of *P*. *crassila* were described by Jewett [17] as a “pair of large subanal lobes that curve evenly upwards extending from median, posterior area”, while for Froehlich [16] the paraprocts of *P*. *klapaleki* were considered “long, almost straight in ascending part, apical part narrow, end rounded”. By analyzing the material studied by Froehlich [16] and Fig 3A in the same study, as well as the photos of type series of *P*. *crassila*, we did not find enough morphological characters to support their status as different from each other. In addition, these two species were sampled in the same locality, that is, in the municipality of Nova Teutônia, Santa Catarina state, Brazil. Thus, the evidence is adequate to propose herein the synonymy of *P*. *crassila* under *P*. *klapaleki*.

#### Geographical distribution

The type locality of *P*. *klapaleki* is the municipality of Petrópolis, Rio de Janeiro state, Brazil [7]. This species was also recorded in Santa Catarina state (material determined by Froehlich in MZSP in the same locality as the *P*. *crassila* materials studied by Jewett [17]), and at the Serra do Mar (Coastal Mountains) and Serra da Mantiqueira (Mantiqueira Mountains) between São Paulo and Rio de Janeiro states [12, 16]. Recently, a nymph was recorded in the Province of Misiones, Argentina [49]. For species geographic records, refer to the map in Fig 3.

***Paragripopteryx dasalmas*** Duarte, Calor and Bispo **sp. nov.** [*urn*:*lsid*:*zoobank*.*org*:*act*:*C480D616-8C26-4FFA-AEEA-46FBC5BDA504*]

(Fig 5A–5G)

**Fig 5:**
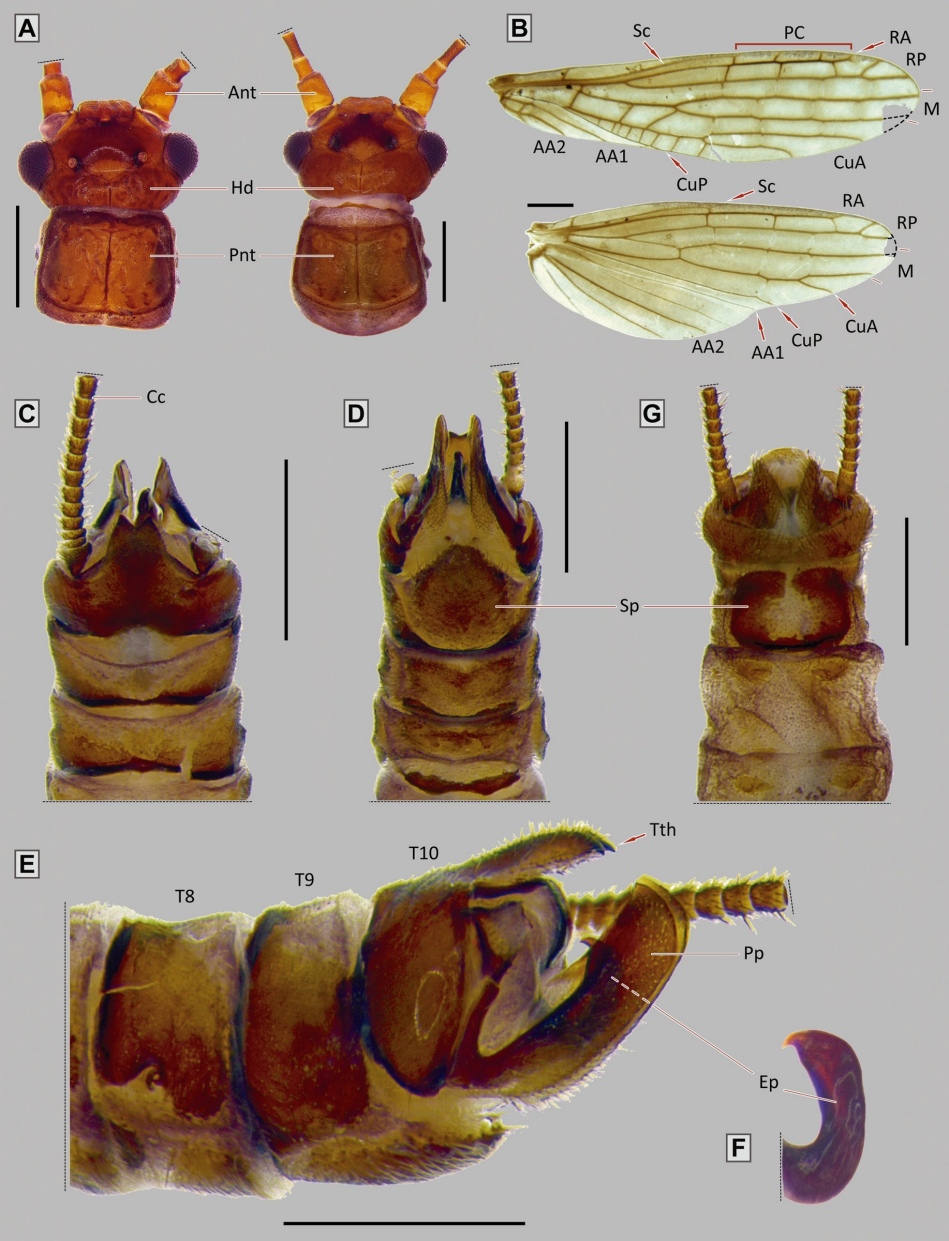
*Paragripopteryx dasalmas* sp. nov. **(A)** Left–holotype adult male, head and pronotum in dorsal view; right–adult female, head and pronotum in dorsal view. **(B)** Male fore- and hind wings. **(C**, **D**, **E)** Male terminalia in dorsal, ventral and lateral views, respectively. **(F)** Detail of the epiproct. **(G)** Female terminalia in ventral view (Scale bars: A, B, C, D: 0.5 mm; E, G: 0.4 mm).

*Material examined*. ***Type material***: Holotype, male, BRAZIL: Bahia, Wenceslau Guimarães, EEWG, Serra Grande Stream, waterfall, S13° 35’34”, W39°42’52”, 10.x.2010, light pan trap (*A*.*R*. *Calor*) (MZSP).–Paratypes: same data as holotype, except: 1 male, 1 female (MZSP); same data, except: 09.x.2010, 1 male, 4 females (MZSP); same data, except: 06.ix.2013, 1 female (*A*.*R*. *Calor*, *T*. *Duarte and E*.*S*. *Dias*) (MZSP); same data, except: 07.ix.2013, 2 males, 2 females (UFBA); same data, except: 06.ix.2013, light trap near the headquarter, 1 female (MZSP); same data, except: S13°35’35.4”, W39°42’51.2”, 513m, 06.ix.2013, 1 female (MZSP); same data, except: 07.ix.2013, 1 female (MZSP); same data, except: 08.ix.2013, 10 females (MZSP); same data, except: 08.ix.2013, light trap, 1 male, 1 female (MZSP); same data, except: Patioba River, S13°34’50”, W39°42’17”, 09.x.2010, light pan trap, 2 females (MZSP).

#### Diagnosis

*Adult*: male T10 with a proximal triangular lighter band; T10E with M-shaped apex ending in two teeth. Male paraprocts thick throughout length, apex with ventral curvature, dorsal margin straight, tip triangular. Male epiproct sickle-shaped without teeth on the inner margin, apex pointed. Female subgenital plate square and with rounded corners, central area unpigmented.

#### Description

*Paragripopteryx dasalmas* sp. nov. is a medium-sized species. ***Coloration***: general color brown. ***Head***: brown with a lighter-brown area between paired ocelli and coronal bifurcation, parietalia rough (Fig 5A). Ocelli and eyes black. Labrum light brown. Maxillary palps light brown, 5-segmented; first and fourth segments short, and second, third and fifth longer. Labial palps light brown, 3-segmented. Antennae brown. ***Thorax***: pronotum brown, square with rounded edges and narrower than the head (Fig 5A). ***Legs***: brown. Tibia with a perpendicular suture in the proximal region and two spurs in the distal region. Tarsi brown, legs with tarsomere 1 medium, tarsomere 2 short, and tarsomere 3 long. ***Wings***: forewings brown with darker patterns bordering veins and crossveins, 1–2 pterostigmatic crossveins present, RA unforked, RP and CuA forked. Hind wings with M3+4, near their separation from M1+2, fused to CuA in part of their length, CuA short and forked, 6th anal vein fused to the wing margin (Fig 5B). ***Male abdomen***: brown with slightly lighter band on abdominal terga 1–9. ***Male terminalia***: in dorsal view, T10 dark brown and with a proximal triangular lighter band, T10E medium-length, apex M-shaped ending in two teeth (Fig 5C). In ventral view, subgenital plate brown with rounded base, distal margin triangular, tip slightly rounded (Fig 5D); paraprocts thin, directed to the T10E and with lighter area on the base; epiproct present and dark. In lateral view, paraprocts thick throughout their length; base covered with setae, apex with a ventral curvature, dorsal margin straight, tip triangular (Fig 5E); epiproct hook- or sickle-shaped without denticles on the dorsal margin and with pointed apex (Fig 5F). ***Female abdomen***: abdominal terga 1–9 light brown and mostly membranous. ***Female terminalia***: T10 brown and with rounded apex. St7 with two small and conspicuous spots. Subgenital plate square and with rounded corners; central area unpigmented; paraprocts small, covered with setae, apex truncated (Fig 5G). ***Nymph***: unknown.

*Measurements*. Holotype, male: head width, 0.74 mm; pronotum width, 0.7 mm; pronotum length, 0.6 mm; forewing length, 6.6 mm; hind wing length, 5.2 mm; antennae length, 6.4 mm; 16 cercomeres; paratypes, males (n = 5): head width, 0.65–0.8 mm; pronotum width, 0.64–0.75 mm; pronotum length, 0.55–0.63 mm; forewing length, 6.4–6.9 mm; hind wing length, 5.4–5.8 mm; antennae length, 5–7.1 mm (incomplete in some specimens, maximum length, 7.1 mm); 17–18 cercomeres; females (n = 24): head width, 0.8–0.83 mm; pronotum width, 0.76–0.77 mm; pronotum length, 0.55–0.7 mm; forewing length, 7.6–7.8 mm; hind wing length, 6.5–7 mm; antennae length, 7.85–7.95 mm; 18 cercomeres.

#### Derivatio nominis

The epithet “*dasalmas*” refers to the Das Almas River (Rio das Almas in Portuguese language) in the municipality of Wenceslau Guimarães in Bahia state, Brazil. The name is in apposition.

#### Comments

*Paragripopteryx dasalmas* is the first species of the genus described and formally recorded in the Brazilian Northeast Region. Duarte et al. [52] provided records of nymphs of the genus at the Serra Bonita Reserve, in southern Bahia. However, the above-mentioned study did not make the specific identification of the specimens. The description of *P*. *dasalmas* is based on male and female specimens sampled at the Ecological Station of Wenceslau Guimarães, municipality of Wenceslau Guimarães, Bahia state. The T10E in males of *P*. *dasalmas* partly resembles that of *P*. *merui* [22]; however, in *P*. *merui* this structure is wider and curved, while in *P*. *dasalmas* it is narrower and straight, ending in a pointed apex (Fig 5C). In addition, whereas the paraprocts in *P*. *dasalmas* are thick throughout their length with angulated dorsal margin in the apex (Fig 5E), in *P*. *merui* they are narrow next to the apex, ending in a subapical tooth. The epiproct of both species also differ: while *P*. *dasalmas* has a sickle-shaped epiproct without teeth on the inner margin and a pointed apex (Fig 5F), in *P*. *merui* the epiproct is long and provided with two rows of teeth on the inner margin. Additionally, *P*. *dasalmas* is darker than *P*. *merui*.

#### Geographical distribution

The type locality of *P*. *dasalmas* is the Ecological Station of Wenceslau Guimarães, located in the municipality of Wenceslau Guimarães, Bahia state, Brazil, which is the only locality where the species has been recorded. The Station is inserted in the South Recôncavo River Basin (Das Almas River or Jequié River sub-basin), with a vegetation cover belonging to the phytogeographic domain of the Atlantic Forest. In this region, there is a high number of endemic species, besides the occurrence of endangered species [53]. For species geographic records, refer to the map in Fig 3.

***Paragripopteryx ogum*** Duarte, Calor and Bispo **sp. nov.** [*urn*:*lsid*:*zoobank*.*org*:*act*:*638E7762-0945-4E44-9B4E-9FD8153E18EB*]

(Fig 6A–6E)

**Fig 6:**
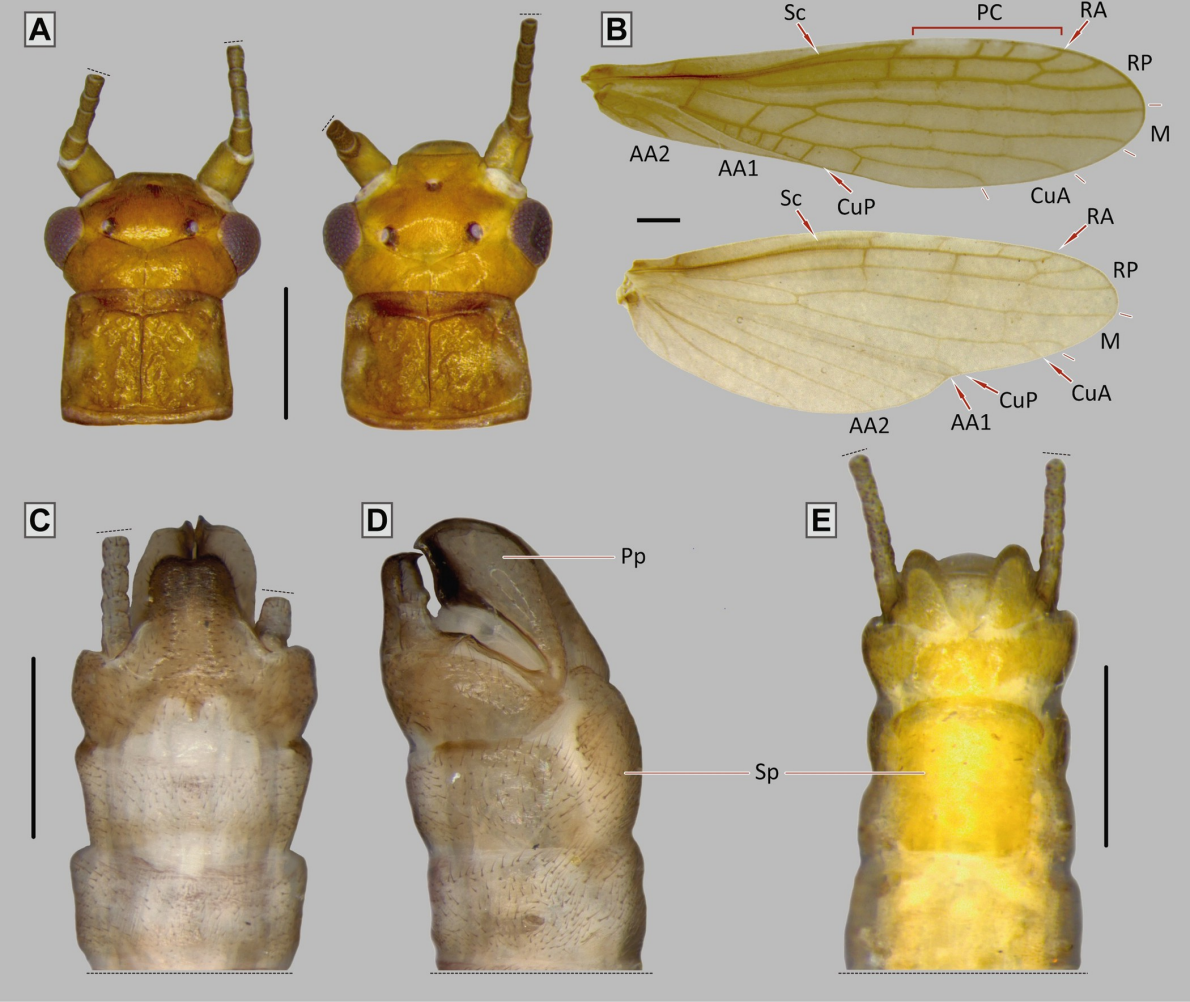
*Paragripopteryx ogum* sp. nov. **(A)** Left–holotype adult male, head and pronotum in dorsal view; right–female head and pronotum in dorsal view. **(B)** Male fore- and hind wings. **(C**, **D)** Male terminalia in dorsal and lateral views, respectively. **(E)** Female, terminalia in ventral view (Scale bars: A, B, E: 0.5 mm; C, D: 0.4 mm).

*Material examined*. ***Type material***: Holotype male, BRAZIL: Santa Catarina, Orleans, Rio Minador, #72, S28°10’24”, W49°24’37”, 12.x–10.xi.2013, Malaise (*L*.*C*. *Pinho*) (MZSP).–Paratypes: same data as holotype, except: 1 female (MZSP); same data, except: #67, 19.viii-12.x.2013, 1 male (MZSP).

#### Diagnosis

*Adult*: male T10 light brown with a proximal lighter M-shaped band; T10E elongate, with distal M-shaped border, end dark provided with two small teeth. Male paraprocts sword-shaped and ventrally curved, apex broader. Male epiproct absent. Female T10 light brown and with rounded apex. Female subgenital plate square and with rounded corners, central area unpigmented.

#### Description

*Paragripopteryx ogum* sp. nov. is a medium-sized species. ***Coloration***: general color light brown to yellowish. ***Head***: brown with a lighter-brown area between paired ocelli and coronal bifurcation (Fig 6A). Ocelli and eyes black. Labrum light brown. Maxillary palps light brown, 5-segmented; first and fourth segments short, and second, third and fifth longer. Labial palps light brown, 3-segmented. Antennae brown. ***Thorax***: pronotum brown, rough, and squarish with subparallel margins (Fig 6A). ***Legs***: brown. Tibia with a perpendicular suture in the proximal region and with two spurs in the distal region. Tarsi brown, legs with tarsomere 1 medium, tarsomere 2 short, and tarsomere 3 long. ***Wings***: forewings membranous with darker patterns bordering veins and crossveins weak; pterostigmatic cell with white patches between pterostigmatic crossveins; RA unforked, RP and CuA forked. Hind wings with M3+4, near their separation from M1+2, fused to CuA in part of their length, CuA short and forked, 6th anal vein fused to the wing margin (Fig 6B). ***Male abdomen***: light brown to ochraceous. ***Male terminalia***: in dorsal view, T10 light brown with a proximal lighter M-shaped band; T10E long, with distal M-shaped border, darker and ending in two small teeth (Fig 6C). In ventral view, subgenital plate ochraceous, apex rounded. In lateral view, paraprocts sword-shaped and ventrally curved with apex broader (Fig 6D). Epiproct absent. ***Female abdomen***: abdominal terga 1–9 light brown and mostly membranous. ***Female terminalia***: T10 light brown and with rounded apex. Subgenital plate square and with rounded corners, central area unpigmented. Paraprocts small, covered by setae and with rounded apex (Fig 6E). ***Nymph***: unknown.

*Measurements*. Holotype, male: head width, 0.83 mm; pronotum width, 0.65 mm; pronotum length, 0.52 mm; forewing length, 6.8 mm; hind wing length, 5.7 mm; number of cercomeres, 16; paratype, female (n = 1): head width, 0.88 mm; pronotum width, 0.73 mm; pronotum length, 0.53 mm; forewing length, 7.4 mm; hind wing length, 6.4 mm; number of cercomeres, 15.

#### Derivatio nominis

Brazilian culture has a great influence of African culture. The specific name “*ogum*” refers to the shape of male paraprocts, which resembles the shortest form of the sword of the Orisha Ogum (Yoruba: Ògún, Portuguese: Ogum). In African traditions, Ogum is considered the great warrior, the first Orisha to descend into the realm of Ile Aiye (“Earth”), opening the way. The name is in apposition.

#### Comments

*Paragripopteryx ogum* is based on specimens sampled in the municipality of Orleans, Santa Catarina state, Brazil. The T10 in *P*. *ogum* partly resembles that of the male of *P*. *merui*. However, the shape of paraprocts is distinct in these two species: whereas in *P*. *ogum* the distal third of paraprocts is broader (Fig 6D), in *P*. *merui* it is narrow.

#### Geographical distribution

The type locality of *P*. *ogum* is the municipality of Orleans, Santa Catarina state, Brazil, which is the only locality where the species has been recorded. The location site is next to the conservation area of the Serra Furada State Park (PAESF), which protects numerous springs [54]. The relative humidity of the area is high (around 85%) and the average annual rainfall is 1,500 mm [55]. For species geographic records, refer to the map in Fig 3.

#### *Paragripopteryx anga* Froehlich, 1969

(Fig 7A; Fig 8A–8C)

**Fig 7:**
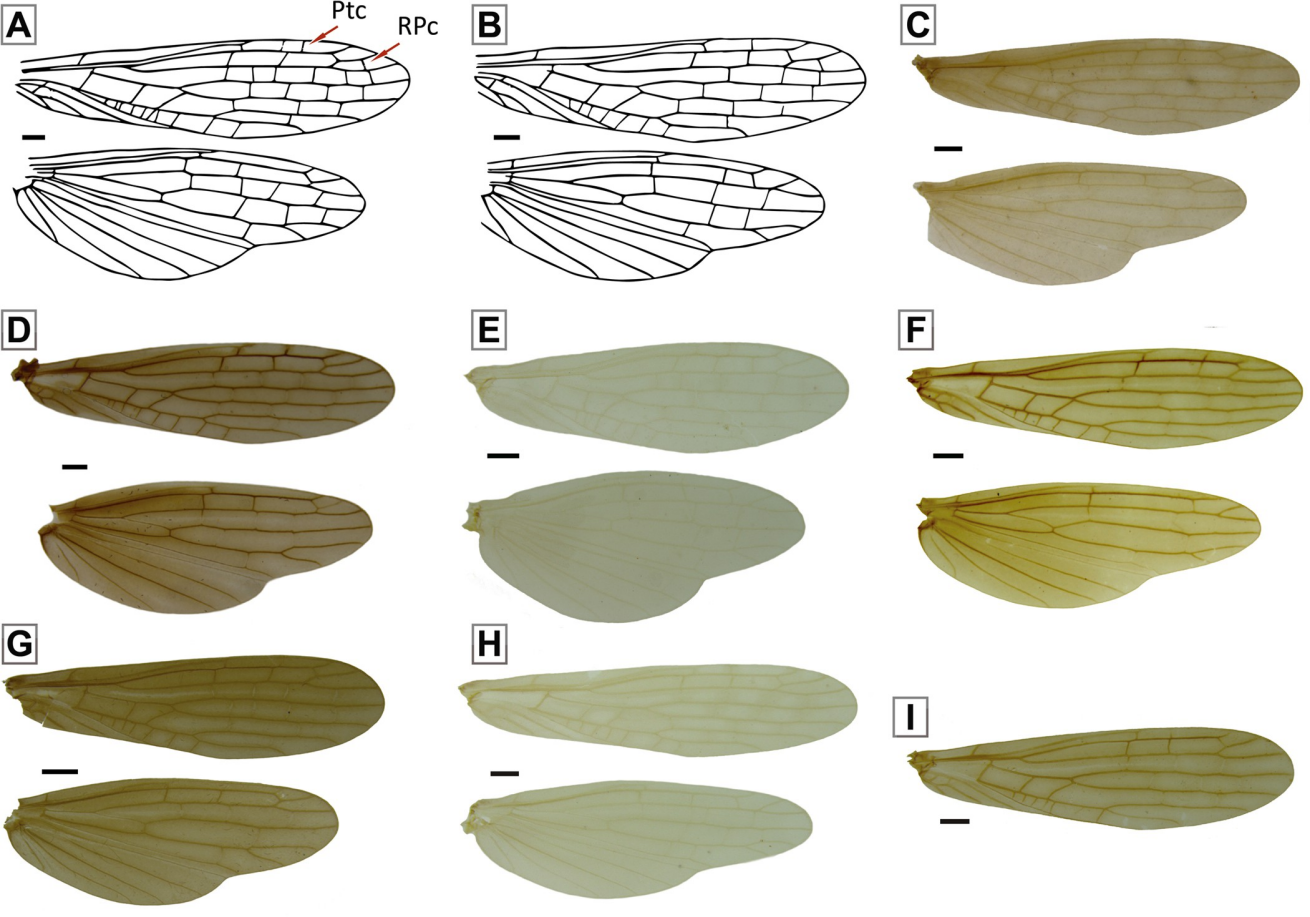
Male wings of the *Paragripopteryx* species. *Paragripopteryx anga*: **(A)** male paratype, fore- and hind wings. *Paragripopteryx blanda*: **(B)** male holotype, fore- and hind wings. *Paragripopteryx delicata*: **(C)** male holotype, fore- and hind wings. *Paragripopteryx egena*: **(D)** male, fore- and hind wings. *Paragripopteryx guardae*: **(E)** male holotype, fore- and hind wings. *Paragripopteryx hamata*: **(F)** male holotype, fore- and hind wings. *Paragripopteryx intervalensis*: **(G)** male specimen from Serra Furada, Santa Catarina state, fore- and hind wings. *Paragripopteryx merui*: **(H)** male holotype, fore- and hind wings. *Paragripopteryx paranapiacabae*: **(I)** male holotype, forewing with pterostigmatic crossvein (Scale bar: 0.5 mm).

**Fig 8:**
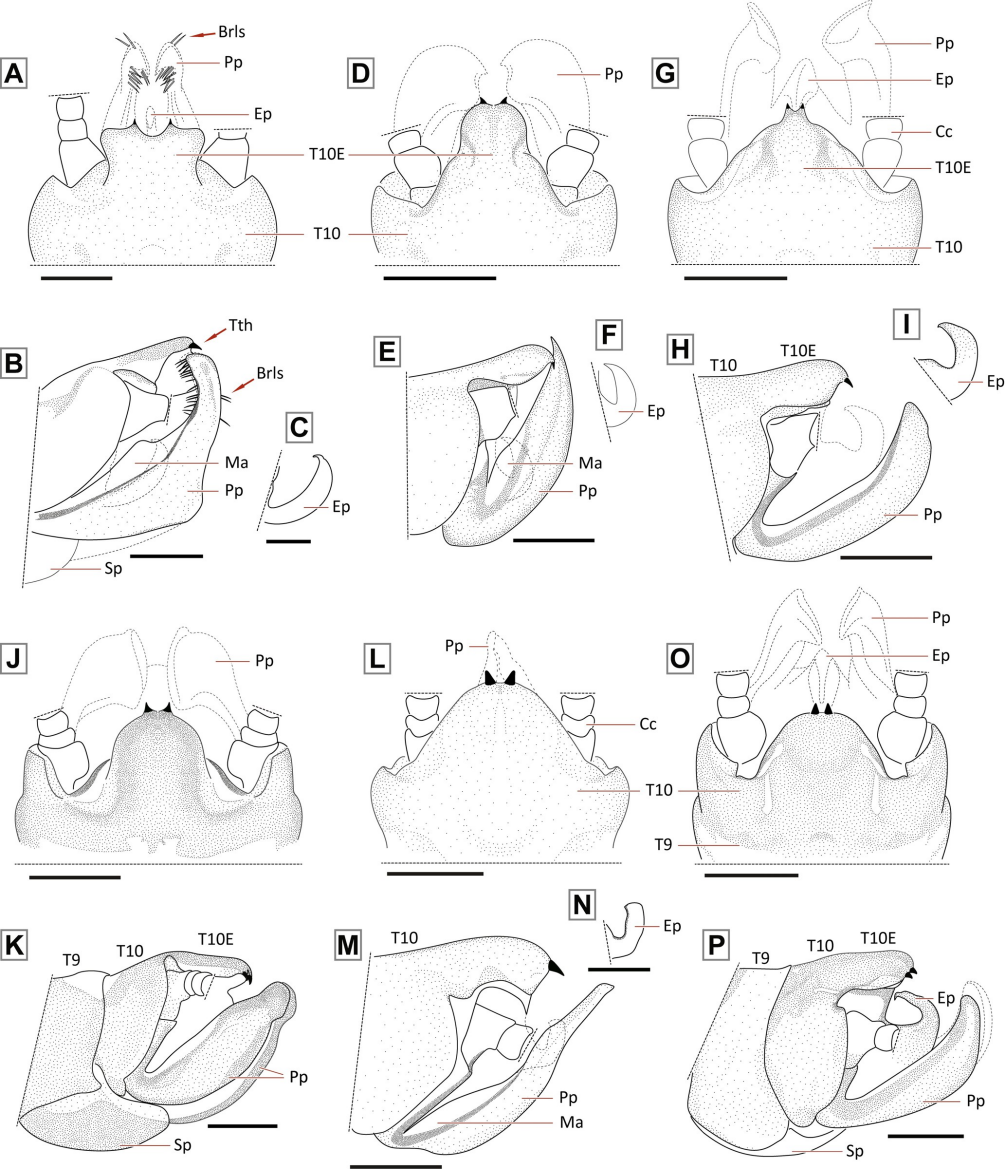
Male terminalia of the *Paragripopteryx* species. *Paragripopteryx anga*: **(A)** male holotype, partial T10 in dorsal view. **(B**, **C)** Male holotype, paraprocts and detail of epiproct in lateral view, respectively. *Paragripopteryx blanda*: **(D)** male specimen from Santa Catarina state, T10 in dorsal view. **(E**, **F)** Paraprocts and epiproct in lateral view, respectively. *Paragripopteryx delicata*: **(G)** male holotype, T10 in dorsal view. **(H**, **I)**: Paraprocts and epiproct in lateral view, respectively. *Paragripopteryx egena*: **(J)** male T10 in dorsal view. **(K)** Paraprocts in lateral view. *Paragripopteryx guardae*: **(L)** male holotype, T10 in dorsal view. **(M**, **N)** Paraprocts and detail of epiproct in lateral view, respectively. *Paragripopteryx hamata*: **(O)** male holotype, T10 in dorsal view. **(P)** Paraprocts and epiproct in lateral view (Scale bar: 0.2 mm).

*Paragripopteryx anga* Froehlich, 1969 [16]—Studies on Neotropical Fauna and Environment, 6(1): 23 (males, females, nymphs, Figs 11–18, 35).

*Paragripopteryx anga* Froehlich, 1994 [22]—Aquatic Insects, 16(4): 228 (review).

*Paragripopteryx anga* Froehlich, 2010 [11]—Illiesia, 6 (12): 135 (catalog).

*Paragripopteryx anga* Bispo and Lecci, 2011 [12]—Annales de Limnologie–International Journal of Limnology, 47: 376–377 (new record).

#### Material examined

Holotype male, BRAZIL: São Paulo, Estação Biológica de Paranapiacaba, 27.iii.1964 (*C*.*G*. *Froehlich*) (MZSP).–Allotype: same data as holotype, except: 28.viii.1963, 1 female.–Paratypes: adults–same data, except: 30.viii.1962, 1 female; same data, except: 12.iv.1963, 1 male, 2 females; same data, except: 5.vii.1963, 1 male, 1 female; same data, except: 19.vii.1963, 1 male (at light); same data, except: 6.viii.1963, 3 males, 1 female; same data, except: 27.viii.1963, 3 males, 2 females (1 female at light); same data, except: 17.ix.1963, 1 male, 3 females; same data, except: 15.x.1963, 2 males, 1 female; same data, except: 5.xi.1963, 1 female; same data, except: 27.xi.1963, 1 male, 2 females; same data, except: 28.xii.1963, 2 males, 3 females; same data, except: 17.i.1964, 1 female; same data, except: 27.iii.1964, 1 male, 1 female; same data, except: 23.iv.1964, 1 male.—Adults emerged in the laboratory: viii.1962, 1 male, 1 female; ix.1962, 3 males, 6 females; iv.1963, 1 male, 1 female; v.1963, 2 females; vii.1963, 9 males, 5 females; viii.1963, 10 males, 10 females; ix.1963, 9 males, 22 females; x.1963, 8 males, 8 females; xi.1963, 3 males, 6 females; v.1964, 1 female; vi.1964, 2 males, 4 females.—Cast exuviae (last nymphal instar): same data, except: 23.vii.1962, 1 male, 1 female; same data, except: 12.iv.1963, 1 female; same data, except: 19.vii.1963, 1 female; same data, except: 27.viii.1963, 2 males, 1 female; same data, except: 15.x.1963, 3 males, 2 females; same data, except: 5.xi.1963, 1 male; same data, except: 27.xi.1963, 3 females; same data, except: 13.xii.1963, 1 female; same data, except: 17.i.1964, 1 male; same data, except: 27.iii.1964, 1 male, 3 females; same data, except: 23.iv.1964, 1 female; same data, except: 20.v.1964, 2 males, 4 females; same data, except: 10.vi.1964, 2 females.

#### Diagnosis

*Nymph*: dorsal row of bristles a little denser on legs; tibiae with both claviform and simple hairs [16]. T10 with two pairs of light spots laterally positioned from the middle region to the posterior margin, the pair of spots can be joined (Fig 18A, 18B in [16]). *Adult*: generally brownish to ochraceous species (gray to brownish gray in life, [16]). Male forewings with pterostigmatic crossveins (Fig 7A) and with a light line present in both wings between RA-RP and M, inconspicuous in lighter specimens. Male T10E spatulate, ending in two teeth separated by a rounded incision (Fig 8A). Male paraprocts elongate, basally thick, mid-to-distal part narrower, end rounded; distal region provided with black rod-like setae (Fig 8B). Male epiproct hook-shaped without denticles on the inner margin (Fig 8C). Female subgenital plate incised in distal edge, median area lighter (Fig 15 in [16]); female paraprocts triangular and pointed.

#### Comments

**Fig 9:**
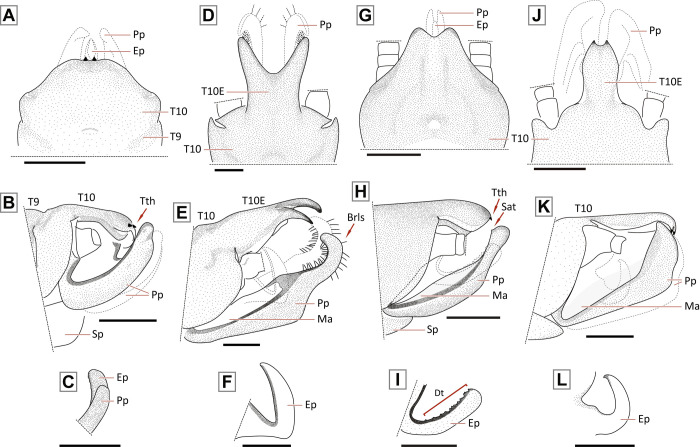
Male terminalia of the *Paragripopteryx* species. *Paragripopteryx intervalensis*: **(A**, **B)** male specimen from Serra Furada, Santa Catarina state, terminalia in dorsal and lateral views, respectively. *Paragripopteryx intervalensis*: **(C)** detail of paraproct and epiproct length from species sampled at the Intervales State Park, São Paulo state. *Paragripopteryx kapilei*: **(D)** male from Santa Catarina state, terminalia in dorsal view. *Paragripopteryx kapilei*: **(E)** paratype male, terminalia in lateral view. *Paragripopteryx kapilei*: **(F)** detail of male epiproct in lateral view. *Paragripopteryx merui*: **(G**, **H)** male, terminalia in dorsal and lateral views, respectively. *Paragripopteryx merui*: **(I)** detail of male epiproct in lateral view. *Paragripopteryx paranapiacabae*: **(J**, **K)** holotype and paratype adult male, terminalia in dorsal and lateral views, respectively. *Paragripopteryx paranapiacabae*: **(L)** detail of male epiproct in lateral view (Scale bar: 0.2 mm).

Male holotype of *P*. *anga* is in a good state of conservation, except for the thorax segments, which are crushed. It is possible to observe the main characters of holotype terminalia. The description of *P*. *anga* by Froehlich [16] is detailed and based on several males, females and nymphs. Froehlich [16] supplied figures of female wings, male and female terminalia, mouthparts and T10 of nymphs, and eggs (Figs 11–18, 35 in [16]). He also discussed the light spot pattern on T10 of advanced nymphs of this species. In his study, he compared *P*. *anga* with *P*. *klapaleki* and *P*. *blanda*–the only *Paragripopteryx* species described at the time. *P*. *anga* can be distinguished from other species of the genus by the male spatulate T10E and the incised subgenital plate of the females. The general shape of the male paraprocts of *P*. *anga* resembles that of *P*. *kapilei*, which has black rod-like setae in their distal region (Figs 8B and 9E). However, *P*. *anga* can be distinguished by its shorter and spatulate T10E (Fig 8A).

#### Geographical distribution

The type locality of *P*. *anga* is the Paranapiacaba Biological Station, São Paulo state, Brazil (around 800 m a.s.l.) [16]. Specimens were also sampled at the Boracéia Biological Station (around 900 m a.s.l.) [22] and the Intervales State Park (around 1,100 m a.s.l.) [12], both located in the higher elevation of the Atlantic Forest in São Paulo state, Brazil. For species geographic records, refer to the map in Fig 3.

### *Paragripopteryx baratinii* Benedetto, 1983, *nomen dubium*

*Paragripopteryx baratinii* Benedetto, 1983 [14]—Studies on Neotropical Fauna and Environment, 18(1): 19–23 (males, female, Figs 1–7).

*Paragripopteryx baratinii* Froehlich, 2010 [11]—Illiesia, 6(12): 135 (catalog).

#### Comments

Benedetto [14] described *P*. *baratinii* based on three male and a female specimens (a male holotype and a female paratype deposited at the UDELAR, Uruguay (Figs 1–7 in [14]), and two male paratypes deposited at the Max-Planck Institute, Germany). The type series of *P*. *baratinii* is currently lost. The original description of this species is useless for our morphological dataset. Therefore, we did not consider this species in our analysis. As highlighted by Froehlich [22], in Figs 2–4 of Benedetto’s [14] description of *P*. *baratinii* there seems to be a posterior sclerite in the T10, an uncommon character in *Paragripopteryx* and other Gripopteryginae that is generally observed in genera of Antarctoperlinae, Leptoperlinae and Zelandoperlinae. Thus, we consider that *P*. *baratinii* has doubtful morphological status and propose that it be considered a *nomen dubium*. We expect that future collections in Uruguay will provide new specimens of *P*. *baratinii*, allowing the designation of a neotype.

#### Geographical distribution

The type locality of *P*. *baratinii* is Aguas Blancas, Department of Lavalleja, Uruguay [14]. For species geographic records, refer to the map in Fig 3.

#### *Paragripopteryx blanda* Froehlich, 1969

(Fig 7B, 8D–8F and 10A)

**Fig 10:**
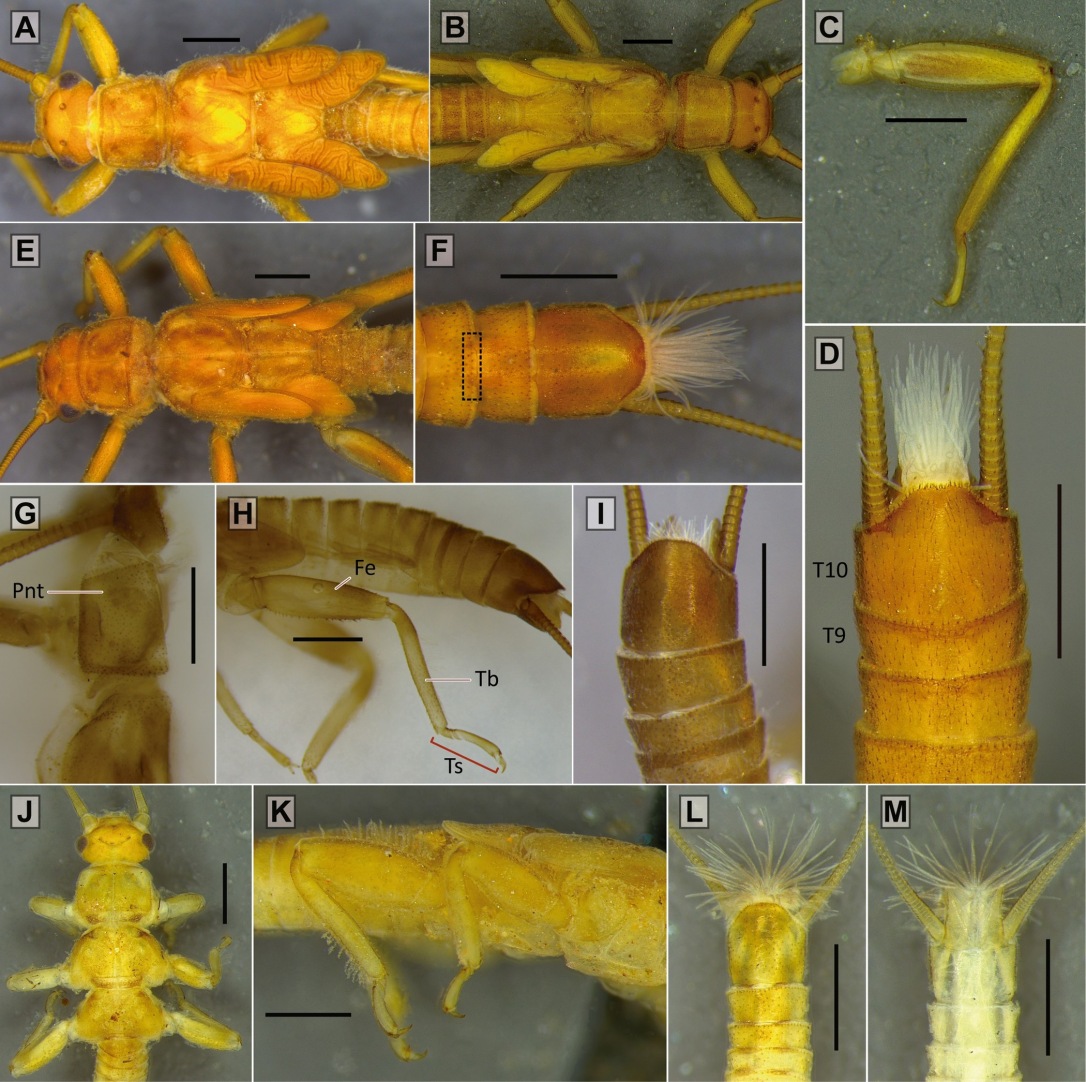
Nymphs of the *Paragripopteryx* species. *Paragripopteryx blanda*: **(A)** body in dorsal view, detail of thorax and legs. *Paragripopteryx delicata*: **(B**, **C**, **D)** body in dorsal view, hindleg and distal abdomen, respectively. *Paragripopteryx guardae*: **(E**, **F**:**)** head and thorax in dorsal view and distal abdomen in dorsal view, respectively. *Paragripopteryx intervalensis*: **(G**, **H**, **I)** nymphal exuviae, pronotum in dorsal view, detail of leg, and T10, respectively. *Paragripopteryx kapilei*: **(J**, **K**, **L**, **M)** young nymph, head and thorax in dorsal view, details of mid- and hindlegs, and abdomen in dorsal and ventral views, respectively. (Scale bar: 0.5 mm).

*Paragripopteryx blanda* Froehlich, 1969 [16]—Studies on Neotropical Fauna and Environment, 6(1): 26–27 (males, females, nymphs, Figs 19–25).

*Paragripopteryx blanda* Froehlich, 1994 [22]—Aquatic Insects, 16(4): 228 (review).

*Paragripopteryx blanda* Froehlich, 2010 [11]—Illiesia, 6(12): 135 (catalog).

*Paragripopteryx blanda* Bispo and Froehlich, 2011 [12]—Annales de Limnologie–International Journal of Limnology, 47: 377 (new record).

#### Material examined

Holotype male, BRAZIL: São Paulo, Estação Biológica de Paranapiacaba, 17.ix.1963 (*C*.*G*. *Froehlich*) (MZSP).–Allotype: same data as holotype, except: 27.iii.1964, 1 female.–Paratypes: adults collected: same data, except: 9.viii.1962, 1 female; same data, except: 30.viii.1962, 1 female; same data, except: 6.viii.1963, 1 male (at light); same data, except: 17.ix.1963, 2 males; same data, except: 15.x.1963, 1 male; same data, except: 27.xi.1963, 2 males, 1 female; same data, except: 13.xii.1963, 1 male (at light), 1 female; same data, except: 28.xii.1963, 1 female; same data, except: 17.i.1964, 2 males, 1 female; same data, except: 29.ii.1964, 1 male; same data, except: 27.iii.1964, 1 male, 2 females; same data, except: 23.iv.1964, 1 female; same data, except: 20.v.1964, 1 male, 2 females.–Adults emerged in the laboratory: iv.1963, 1 female; vii.1963, 1 male, 2 females; viii.1963, 1 male, 1 female; x.1963, 2 males; v.1964, 1 male, 1 female.–Cast exuviae (last nymphal instar): same data, except: 19.vii.1963, 2 females; same data, except: 5.xi.1963, 1 female; same data, except: 27.xi.1963, 1 male; same data, except: 13.xii.1963, 1 male, 1 female; same data, except: 28.xii.1963, 1 female; same data, except: 17.i.1964, 1 male; same data, except: 27.iii.1964, 1 male, 1 female; same data, except: 23.iv.1964, 1 male, 1 female.—***Other material***: BRAZIL: Santa Catarina, Serra Furada, 1 male (*L*.*C*. *Pinho*) (MZSP) (*new record*).

#### Diagnosis

*Nymph*: dorsal row of long hairs of femora and tibiae dense (Fig 10A); female paraprocts with rounded apex; male paraprocts with an apical process (Fig 24 in [16]). *Adult*: general color dark brown (dark brownish-gray in life, [16]). Male forewings with pterostigmatic crossveins (Fig 7B), sometimes bordered by darker spots; pterostigmatic cell occasionally violet and with a light line present in both wings between RA-RP and M. Male paraprocts robust in basal half, narrowing towards apex, and ending in a sharp point (Fig 8E). Male epiproct hook-shaped, without denticles (Fig 8F). Female subgenital plate rounded; apex of paraprocts truncated (Fig 23B in [16]).

#### Comments

**Fig 11:**
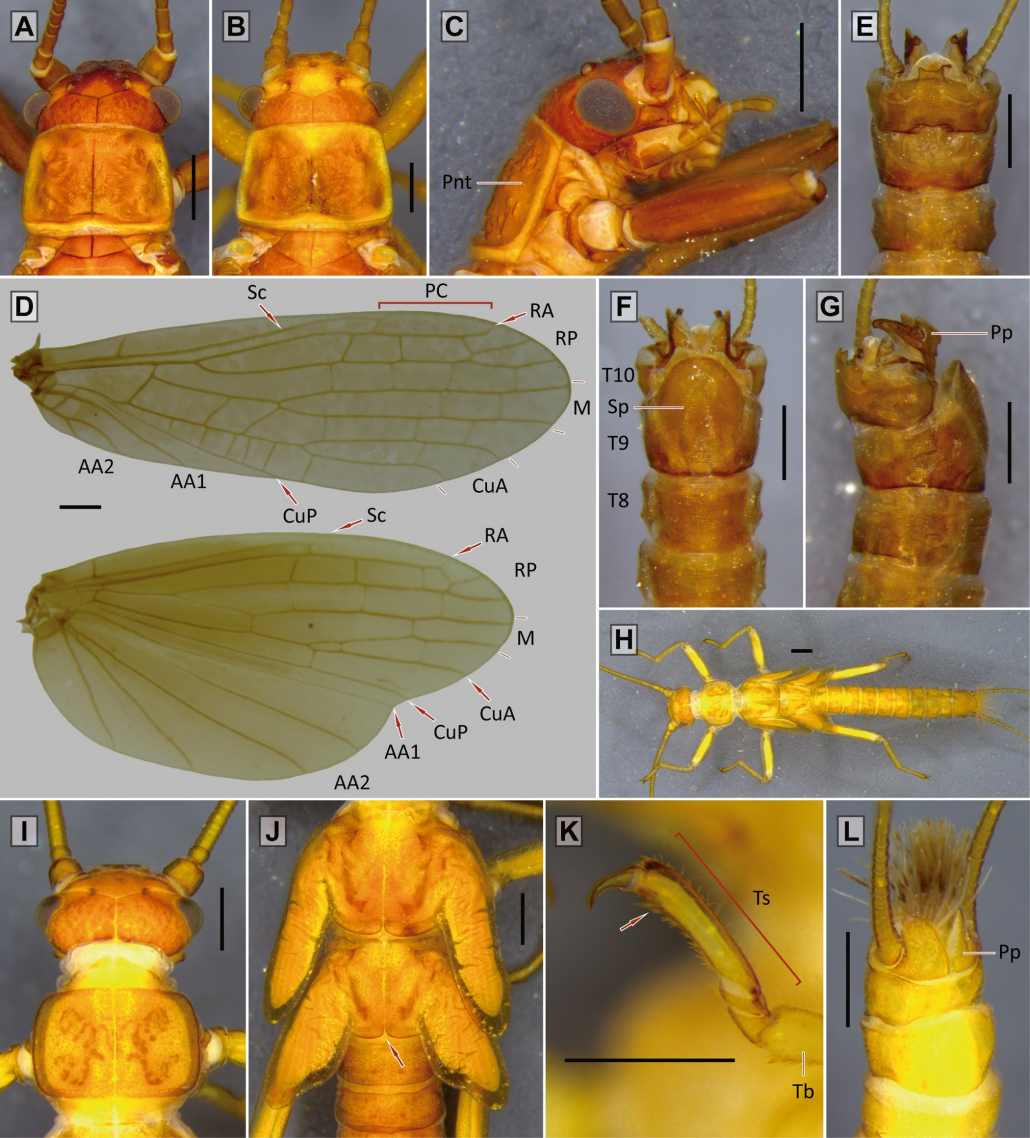
*Paragripopteryx munoai* Benedetto, 1969. **(A)** Paratype adult male, head and pronotum in dorsal view. **(B)** Paratype adult female, head and pronotum in dorsal view. **(C)** Adult male, head and pronotum in lateral view. **(D)** Male fore- and hind wings. **(E**, **F**, **G)** Male terminalia in dorsal, ventral, and lateral views, respectively. **(H)** Nymph, body in dorsal view. **(I)** Nymph, head and pronotum in dorsal view. **(J)** Nymph, meso- and metanotum in dorsal view. **(K)** Nymph, detail of tarsi. **(L)** Nymph, abdominal ends in latero-ventral view (Scale bars: A–L: 0.5 mm).

Froehlich [16] described the male, female and nymph. We examined the type material of this species and concluded that it is in a good state of conservation. The description of *P*. *blanda* by Froehlich [16] is complete, with figures of male wings, male and female terminalia, mouthparts and T10 of nymphs (Figs 11–25 in [16]). At the time, he also compared the male of this species with the male of *P*. *klapaleki*. In our examination, we found a more pronounced similarity of the T10 of *P*. *blanda* with that of *P*. *egena*, except for the conspicuous lateral sclerite in the latter. Additionally, these two species can be distinguished by the distal shape of the paraprocts, which are narrower and pointed in *P*. *blanda* and broadly rounded in *P*. *egena*, as well as by the presence of epiproct in *P*. *blanda*. As mentioned by Froehlich [16], advanced nymphs of *P*. *blanda* are readily distinguished from those of *P*. *klapaleki* by their slenderer body, shorter paraprocts with rounded apex and the uniform color of T10.

#### Geographical distribution

The type locality of *P*. *blanda* is the Paranapiacaba Biological Station, São Paulo state, Brazil [16]. Material was also sampled at the Boracéia Biological Station [22] and the Intervales State Park [12], both in São Paulo state, Brazil. We provide a new record for the Serra Furada State Park (PAESF), Santa Catarina state, Brazil. For species geographic records, refer to the map in Fig 3.

### Paragripopteryx delicata Froehlich, 1994

(Fig 7C; Fig 8G–8I; Fig 10B–10D)

*Paragripopteryx delicata* Froehlich, 1994 [22]—Aquatic Insects, 16(4): 229–231 (males, females, nymphs, Figs 1–5).

*Paragripopteryx delicata* Froehlich, 2010 [11]—Illiesia, 6(12): 135 (catalog).

#### Material examined

Holotype male, BRAZIL: São Paulo, Campos do Jordão, Parque Estadual, 1600 m, 26.xi.1986 (MZSP).–Paratypes: same data as holotype, except: 11.xi.1985, 1 female (*C*.*G*. *Froehlich and L*.*G*. *Oliveira*); same data, except: 7.i.1986, 1 male, 2 females; same data, except: 3.x.1986, 1 female; same data, except: 30–31.x.1986, 1 female; same data, except: 21–23.xii.1986, 2 females; same data, except: 25.iii.1987, 1 female; same data, except: 17–20.xi.1987, 3 males, 5 females; same data, except: 14–16.xii.1987, 1 male, 3 females.—***Other material***: BRAZIL: São Paulo, Salesópolis, Estação Biológica de Boracéia, 850m, 12.x.1974, 1 male, 1 female (*C*.*G*. *Froehlich and O*. *Froehlich*) (MZSP); same data, except: 16.xi.1974, 1 male, 1 female + exuviae; same data, except: 2.xi.1975, 1 male (*S*.*A*. *Vanin*); same data, except: 22–24.x.1982, 2 females; same data, except: 22.ii.1990, 1 female (*C*.*G*. *Froehlich and L*.*G*. *Oliveira*); same data, except: 10–13.xi.1990, 1 male, 3 females (*C*.*G*. *Froehlich*, *L*.*G*. *Oliveira and M*.*J*.*N*. *Ferreira*); 17.ix.1965, 1 nymph (*C*.*G*. *Froehlich and L*.*G*. *Oliveira*); same data, except: 16.xi.1974, 2 nymphs; same data, except: 22.ix.1987, 3 nymphs (*C*.*G*. *Froehlich*, *O*. *Froehlich and M*. *Schweiger*). BRAZIL: São Paulo, Campos do Jordão, Umuarama, 29.i.1959, 1 nymph (*C*.*G*. *Froehlich and L*.*G*. *Oliveira*) (MZSP); same data, except: Parque Estadual, 9.xi.1985, 1 nymph; same data, except: 7.i.1986, 11 nymphs.

#### Diagnosis

*Nymph*: dorsal row of long bristles on femora and tibiae. Wing pads with normal hairs, claviform bristles absent (Fig 10B and 10C). Apex of T10 slightly truncate, surface covered with normal bristles, claviform bristles restricted to distal margin (Fig 10D). Male nymphs with paraprocts upturned, apex pointed; female nymphs with paraprocts apically oval and provided with thin subapical extension appressed to the base of cerci; claviform hairs elongate present on apical portion of paraprocts [22]. *Adult*: generally brown species. Forewings with dark-bordered on either side of the crossveins, pterostigmatic crossveins present (Fig 7C). Male T10E triangular-shaped ending in two small teeth, remarkably close together (Fig 8G). Male paraprocts broad, which may be subapically broader (Fig 8H), apex somewhat pointed (Figs 3, 4 in [22]). Male epiproct large, upturned and hook-shaped without row of denticles on the inner margin (Fig 8I). Female subgenital plate with a pair of lateral sclerotizations, apex broad, margin almost straight. Female paraprocts truncate in ventral view (Fig 5 in [22]).

#### Comments

Type material of *P*. *delicata* is in a good state of conservation, being possible to observe the main characters of terminalia. The description of *P*. *delicata* by Froehlich [22] is detailed and based on several male, female, and nymph specimens. Although he did not provide illustrations of the nymphs of this species, the male and female were illustrated in his study. As discussed by the author, *P*. *delicata* has some morphological characters similar to those of *P*. *blanda*, mainly in relation to the T10 and paraprocts. However, the male T10E of *P*. *delicata* is triangular-shaped with a small distal process, while in *P*. *blanda* this structure is prominent. In addition, the male paraprocts are broad in *P*. *delicata* (Fig 8H), whereas in *P*. *blanda* they narrow towards the apex, ending in a sharp point (Fig 8E). In our analysis, these two species appeared in a collapsed Clade H close to *P*. *egena* (Fig 2).

#### Geographical distribution

The type locality of *P*. *delicata* is the Campos do Jordão State Park, located in the municipality of Campos do Jordão, São Paulo state, Brazil [22]. The area has an average altitude of 1,700 m a.s.l., and the annual average temperature is 16–18°C [56]. Material was also sampled at the Boracéia Biological Station, São Paulo state, Brazil [22]. For species geographic records, refer to the map in Fig 3.

### Paragripopteryx egena Froehlich, 1994

(Fig 7D; Fig 8J–8K)

*Paragripopteryx egena* Froehlich, 1994 [22]—Aquatic Insects, 16(4): 237–239 (males, females, Figs 26–30).

*Paragripopteryx egena* Froehlich, 2010 [11]—Illiesia, 6(12): 136 (catalog).

*Paragripopteryx egena* Bispo and Lecci, 2011 [12]—Annales de Limnologie–International Journal of Limnology, 47: 377.

*Paragripopteryx egena* Gonçalves, Novaes and Salles, 2017 [51]—Zootaxa, 4291(3): 568 (new record).

*Material examined*. Holotype male, BRAZIL, São Paulo, Ribeirão Grande, Fazenda Intervales, 18.xi.1992 (*C*.*G*. *Froehlich and C*.*M*. *Polegatto*) (MZSP).—***Other material***: BRAZIL: Santa Catarina, Orleans, Minador Stream, #76, S28°10’28”, W49°24’36”, Malaise, 10.xi–13.xii.2013, 1 male (*L*.*C*. *Pinho*) (MZSP) (*new record*); BRAZIL: Santa Catarina, Urubici, PARNA São Joaquim, PPBioMA, M1PR1 TN1200, S28°08’36”, W49°38’13”, Malaise, 23.viii–05.ix.2014, 1 male (MZSP) (*new record*); BRAZIL: Santa Catarina, Urubici, #82, 09.xi–12.i.2014, 2 males, 6 females (MZSP) (*new record*); BRAZIL: Santa Catarina, Urubici, RPPN Portal das Nascentes, #64, S28°03’12”, W49°22’27”, Malaise, 19.viii–12.x.2013, 1 male, 2 females (MZSP) (*new record*); BRAZIL: Santa Catarina, Urubici, RPPN Portal das Nascentes, #68, S28°03’02”, W49°22’44”, Malaise, 12.x–09.xi.2013, 1 male, 3 females (MZSP) (*new record*).

#### Diagnosis

*Adult*: general color brown to dark brown. Male forewings with pterostigmatic crossveins (Fig 7D). Male T10 with a pair of indentations on the anterior margin; T10E projected backwards, ending in two small teeth (Fig 8J). Male lateral sclerites in T10 well-separated from the central sclerite in dorsal view (Fig 8J). Male paraprocts thick, apical region narrow and apex rounded in oblique lateral view (Fig 8K); epiproct sclerotized absent [22]. Female subgenital plate may be divided by a median pale band; emargination of apical margin from broadly to shallowly rounded (Figs 29–30 in [22]). Female paraprocts normal, with apex slightly truncate [22].

#### Comments

The type material of *P*. *egena* is in a good state of conservation. Froehlich [22] described male and female adults of *P*. *egena* in detail. So far, the nymphs of this species are unknown. We provide herein photography of male wings and new illustrations of terminalia. The main character that distinguishes *P*. *egena* from most congeners is the absence of a sclerotized epiproct (also absent in *P*. *munoai* and *P*. *ogum* sp. nov.). In our analysis, *P*. *egena* appeared in a collapsed clade with *P*. *delicata* and *P*. *blanda* (Fig 2).

#### Geographical distribution

The type locality of *P*. *egena* is the Intervales Farm (currently known as the Intervales State Park), São Paulo state, Brazil [22]. Gonçalves et al. [51] also reported the species at the Augusto Ruschi Biological Reserve, Espírito Santo state, Brazil. We provide herein new records of this species for the municipalities of Orleans and Urubici, both in Santa Catarina state, Brazil. For species geographic records, refer to the map in Fig 3.

### Paragripopteryx guardae Froehlich, 1994

(Figs 7E, 8L–8N, 10E and 10F)

*Paragripopteryx guardae* Froehlich, 1994 [22]—Aquatic Insects, 16(4): 231–232 (males, females, nymphs, Figs 6–12).

*Paragripopteryx guardae* Froehlich, 2010 [11]—Illiesia, 6(12): 136 (catalog).

*Material examined*. Holotype male, BRAZIL: São Paulo, Campos do Jordão, Parque Estadual, 17.x.1985 (*C*.*G*. *Froehlich and L*.*G*. *Oliveira*) (MZSP).–Paratypes: same data as holotype, except: 17.x.1985, 3 males, 4 females, 7 exuviae (4 males, 3 females), 18 nymphs; same data, except: 9.xi.1985, 2 nymphs.

#### Diagnosis

*Nymph*: numerous claviform bristles on wing pads (Fig 10E). Leg fringe weak, hind femora with stout and short bristles along ventral ridges (Fig 12 in [22]). T10 covered with normal bristles and some claviform ones; posterior margin with numerous claviform bristles (Fig 10F). Female paraprocts long and triangular; male paraprocts long and with claviform bristles elongate present near apex [22]. *Adult*: general color ochraceous to yellowish. Male forewings pale brown, pterostigmatic crossveins present (Fig 7E). Male lateral sclerites in T10 weak; male T10E projected backwards, half-elliptical, ending in two small teeth (Fig 8L). Male paraprocts thin, apical region narrower; epiproct small (Fig 8M and 8N). Female subgenital plate with lateral sclerotizations, apex notched. Female paraprocts thin (Fig 9 in [22]).

#### Comments

The type material of *P*. *guardae* is in a good state of conservation. Froehlich [22] provided detailed description of *P*. *guardae* with illustrations of male and female terminalia, and details of femur and paraprocts of nymphs. In his discussion, he compared *P*. *guardae* with *P*. *klapaleki*. Both species differ in relation to T10E, which is larger and rounded in males of *P*. *guardae*. Additionally, the nymphs of *P*. *guardae* differ from those of *P*. *klapaleki* by the femur bearing stout and short bristles on its ventral side [22].

#### Geographical distribution

The type locality of *P*. *guardae* is the Campos do Jordão State Park, São Paulo state, Brazil [22], which is the only locality where this species was sampled. For species geographic records, refer to the map in Fig 3.

### Paragripopteryx hamata Froehlich, 1994

(Fig 7F, 8O and 8P)

*Paragripopteryx hamata* Froehlich, 1994 [22]—Aquatic Insects, 16(4): 234–236 (males, females, Figs 18–21).

*Paragripopteryx hamata* Froehlich, 2010 [11]—Illiesia, 6(12): 136 (catalog).

*Paragripopteryx hamata* Bispo and Lecci, 2011 [12]—Annales de Limnologie—International Journal of Limnology, 47: 377 (new records).

*Material examined*. Holotype male, BRAZIL: São Paulo, Salesópolis, Estação Biológica de Boracéia, 850 m, 12.xi.1990 (*C*.*G*. *Froehlich*, *L*.*G*. *Oliveira and M*.*J*.*N*. *Ferreira*) (MZSP).–Paratypes: same data as holotype, except: 26.i.1974, 1 female (*C*.*G*. *Froehlich and I*.*R*. *Ball*); same data, except: 5–22.xii.1987, 1 male (*O*. *Froehlich and M*. *Schweiger*).

#### Diagnosis

*Adult*: general color dark brown. Male forewings with paler pterostigmatic cell, crossveins present (Fig 7F). Male T10 with a pair of conspicuous longitudinal bands not sclerotized in distal part (like clefts, but not open) (Fig 8O); T10E projected backwards, distal margin rounded, ending in two small teeth remarkably close. Male paraprocts relatively narrow, apex rounded; epiproct hook-shaped and without row of denticles on the inner margin (Fig 8P). Female with subgenital plate relatively long and medially depigmented, apex rounded (Fig 21 in [22]).

#### Comments

We examined the type material of *P*. *hamata* and concluded that it is in a good state of conservation. Froehlich [22] provided good illustrations of male and female terminalia (Figs 18–21 in [22]) and compared *P*. *hamata*, at that time, with *P*. *blanda*. The male T10 with a pair of conspicuous bands not sclerotized in the distal part remarkably distinguish this species from other congeners. In our analysis, *P*. *hamata* is in a polytomy along with *P*. *paranapiacabae* and Clade G (Fig 2). Nymphs are unknown.

#### Geographical distribution

The type locality of *P*. *hamata* is the Boracéia Biological Station, São Paulo state, Brazil [22]. Material was also sampled at the Intervales State Park, São Paulo state, Brazil [12]. For species geographic records, refer to the map in Fig 3.

### *Paragripopteryx intervalensis* Bispo and Lecci, 2011

(Figs 7G, 9A–9C and 10G–10I)

*Paragripopteryx intervalensis* Bispo and Lecci, 2011 [12]—Annales de Limnologie—International Journal of Limnology, 47: 377–378 (male, Figs 2–4).

*Paragripopteryx intervalensis* Gonçalves, Novaes and Salles, 2017 [51]—Zootaxa, 4291(3): 568–569 (female, Figs 7–9).

*Material examined*. Holotype male, BRAZIL: São Paulo, Iporanga, Parque Estadual Intervales, Chico Paes, 31.x.2002 (*A*.*S*. *Melo*, *R*.*G*. *Alves and P*.*C*. *Bispo*) (MZSP).—***Other material***: BRAZIL: São Paulo, Iporanga, Parque Estadual Intervales, Rib. Poços Altos, [1030C], 18.xi.1992, 1 male (*C*.*G*. *Froehlich and C*.*M*. *Polegatto*) (MZSP); BRAZIL, Espírito Santo, Santa Teresa, Reserva Biológica Augusto Ruschi, stream along the road, close to small waterfall site S19°53’20.6”, W40°32’41.5”, 803 m, light trap, 08-09.ix.2015, 1 exuviae (*F*.*F*. *Salles*) (UFV); BRAZIL: Santa Catarina, Serra Furada, 1 male (*L*.*C*. *Pinho*) (MZSP) (*new record*); BRAZIL: Santa Catarina, Janela, #28, Malaise, 16.xi–08.i.2013, 2 males (MZSP) (*new record*); BRAZIL: Minas Gerais, São Gonçalo do Rio Abaixo, [BR981117], Córrego, Burucutu, Estação Ambiental de Peti (CEMIG), S19°52.995’, W43°22.757’, 1 male (*H*. *Paprocki*) (MZSP) (*new record*).

#### Diagnosis

*Adult*: general color brown. Male forewings with pterostigmatic crossveins (Fig 7G). Male T10E with apex rounded and two small teeth forming a tiny M-shaped projection (Fig 9A). Male subgenital plate apically rounded (Fig 4 in [12]). Male paraprocts simple, apex rounded (Fig 9B and 9C); epiproct simple, elongate and projected between paraprocts [12], sometimes surpassing this structure (Fig 9C). Female with subgenital plate relatively long and square-shaped (Fig 7 in [51]).

#### Description of nymphal exuviae

General color brown to dark brown (Fig 10G–10I). Thorax provided with claviform bristles; pronotum square, almost trapezoidal, corners rounded (Fig 10G). Femora presenting normal and claviform bristles and with some stout and short bristles along the ventral margin (Fig 10H). Abdominal terga presenting both normal and claviform bristles; distal margin of T10 with numerous claviform bristles (Fig 10I). Paraprocts with rounded apex. Gills short.

#### Comments

Bispo and Lecci [12] described *P*. *intervalensis* based on a single male, while Gonçalves et al. [51] described the female (Figs 7–9 in [51]). Herein, we provide the description of female exuviae from the material described by Gonçalves et al. [51]. The stout and short bristles along the ventral margin of the femur of nymphal exuviae in *P*. *intervalensis* resemble those of *P*. *guardae*. Additionally, our new material shows that the male adult epiproct differs in length from the type material, not exceeding the T10 (Fig 9B). The elongate epiproct partially resembles that found in *Aubertoperla illiesi*.

#### Geographical distribution

The type locality of *P*. *intervalensis* is the Intervales State Park, São Paulo state, Brazil [12]. Later, Gonçalves et al. [51] recorded the species in the municipality of Santa Teresa, Espírito Santo state, Brazil. In this study, we provide new records of this species for the Serra Furada State Park (PAESF), Santa Catarina state, Brazil, and São Gonçalo do Rio Abaixo, Minas Gerais state, Brazil. For species geographic records, refer to the map in Fig 3.

### Paragripopteryx kapilei Bispo and Lecci, 2011

(Figs 9D–9F and 10J–10M)

*Paragripopteryx kapilei* Bispo and Lecci, 2011 [12]—Annales de Limnologie—International Journal of Limnology, 47: 378 (male, Figs 5–7).

*Material examined*. Holotype male, BRAZIL: São Paulo, Iporanga, Parque Estadual Intervales, Córrego Roda D’Água, 02.ii.2000 (*C*.*G*. *Froehlich*, *A*.*S*. *Melo*, *V*.*R*. *Ribeiro and P*.*C*. *Bispo*) (MZSP).–Paratype: same data, except: 10.v.2005, 1 male (*R*. *Mariano*).—***Other material***: BRAZIL: Santa Catarina, Blumenau, Spitzkopf, S27°00’22”, W49°06’28”, [1453], 28.xi.2003, 1 male, 1 nymph + exuviae (*C*.*G*. *Froehlich*, *R*.*W*. *Holzenthal and A*.*R*. *Calor*) (MZSP) (*new record*); Ribeirão do Ouro, S27°00’35”, W49°06’69”, [1293], 700m, 03.iii.1998, 1 nymph (*C*.*G*. *Froehlich*, *R*.*W*. *Holzenthal and H*. *Paprocki*) (*new record*).

#### Diagnosis

*Adult*: general color ochraceous to yellowish. Male forewings with pterostigmatic crossveins. Male T10 with a conspicuous lateral cleft on each side (Fig 9D and 9E); T10E elongate and Y-shaped (Fig 9D). Male paraprocts elongate and with a medially small elevation, distal region before the apex narrow, apex rounded, stout setae present (Fig 9E). Male epiproct hook-shaped without row of denticles on the inner margin (Fig 9F). Female unknown.

#### Description of young nymphs

General color ochraceous to yellowish; head yellowish, antennae ochraceous; pronotum ochraceous with rounded corners; meso- and metanotum ochraceous. Wing pads with numerous stout and short bristles (Fig 10J). Femora robust, with some stout and short bristles; femur and tibia with a dorsal row of hairs (Fig 10K). Abdominal terga yellowish, covered with numerous stout and short bristles, distal margin of terga with a dark spot; posterior margin of T10 rounded, almost truncated, with numerous claviform hairs (Fig 10L). Cerci ochraceous. Gills ochraceous. Ventral side lighter in color. Paraprocts elongate (Fig 10M).

#### Comments

Bispo and Lecci [12] described *P*. *kapilei* based on two male specimens. The type material is in a good state of conservation, being possible the examination of the main characters of wings and terminalia. The description of *P*. *kapilei* by Bispo and Lecci [12] is precise and it presents details of the species terminalia (Figs 5–7 in [12]). We examined a male specimen and nymphs from the municipality of Blumenau, Santa Catarina state, Brazil. The nymphs were reared in the field by Dr. Claudio G. Froehlich, and one of them emerged as a male adult, enabling the species identification. Another young nymph is being described herein. In our phylogenetic analyses, *P*. *kapilei* is closer to *P*. *anga* (Fig 2).

#### Geographical distribution

The type locality of *P*. *kapilei* is the Intervales State Park, São Paulo state, Brazil [12]. In this work, we provide new records of this species for the Spitzkopf mountain in the municipality of Blumenau, Santa Catarina state, Brazil. The area is part of the Serra do Itajaí National Park, a conservation unit along the Serra do Mar covered by the Atlantic Rainforest. For species geographic records, refer to the map in Fig 3.

### Paragripopteryx merui Froehlich, 1994

(Figs 7H and 9G–9I)

*Paragripopteryx merui* Froehlich, 1994 [22]—Aquatic Insects, 16(4): 232 (males, exuviae, Figs 13–17).

*Paragripopteryx merui* Froehlich, 2010 [11]—Illiesia, 6(12): 135 (catalog).

*Paragripopteryx merui* Gonçalves, Novaes and Salles, 2017 [51]—Zootaxa, 4291(3): 570 (male specimen does not belong to *P*. *klapaleki*).

*Material examined*. Holotype male, BRAZIL: São Paulo, Campos do Jordão, Parque Estadual, 20.xi.1987, beating sheet (*C*.*G*. *Froehlich*, *L*.*G*. *Oliveira and M*.*J*.*N*. *Ferreira*) (MZSP).—***Other material***: BRAZIL: São Paulo, Campos do Jordão, Parque Estadual, Córrego Garalhada, Quiosque Viveiro, light, 02.xi.2005, 1 male (*M*.*R*. *Spies*) (MZSP); BRAZIL: Espírito Santo, Parque Nacional do Caparaó, Municipality of Espera Feliz, border between Minas Gerais and Espírito Santo states, Pedra Menina, S20°37’30.3”, W41°49’27.1”, 884m, light trap, 16.ii.2016, 1 male (*F*.*F*. *Salles and his team*) (UFV) (*new record*).

#### Diagnosis

*Nymph*: stout and short bristles on femur (Fig 12 in [22]). Wing pads with claviform and few setiform hairs. Paraprocts triangular-shaped, with an apical rounded projection; claviform hairs subapically present. *Adult*: generally light brown. Male forewings with pterostigmatic crossveins (Fig 7H). Male T10 sclerotized, with a conspicuous light spot in the central area and inconspicuous lateral clefts on each side of T10 (like a cleft, but not open) (Fig 9G); T10E with a median notch, ending in two separated teeth, M-shaped (Fig 9G). Male paraprocts relatively thin, apex narrow and rounded, and with a subapical anterior tooth (Fig 9H); epiproct robust, elongate and with two rows of denticles on the inner margin (Fig 9I). Female unknown.

#### Comments

Froehlich [22] described *P*. *merui* based on male specimens. As proposed by the author, the elongate epiproct provided with irregular teeth is distinctive. The description of *P*. *merui* by Froehlich is detailed, and therefore does not require redescription.

#### Geographical distribution

The type locality of *P*. *merui* is the Campos do Jordão State Park, São Paulo state, Brazil [22]. The male specimen was recorded by Gonçalves et al. [51] at the Caparaó National Park (on the border between Minas Gerais and Espírito Santo states) and identified as *P*. *klapaleki*, belonging to *P*. *merui*. For species geographic records, refer to the map in Fig 3.

### Paragripopteryx munoai Benedetto, 1969

(Fig 11A–11L)

*Jewettoperla munoai* Benedetto, 1969 [13]—Beiträge zur Neotropischen Fauna, 6(2): 145–151 (males, females, nymphs, Figs 1–13).

*Jewettoperla munoai* Benedetto, 1970 [57]—Limnologica, 7(2): 383–389 (Figs 2, 4, 5).

*Paragripopteryx munoai* Zwick, 1973 [20]—Das Tierreich, 94: 210 (comb. nov.).

*Paragripopteryx munoai* Froehlich, 2010 [11]—Illiesia, 6(12): 136 (catalog).

*Material examined*. Holotype male, URUGUAY: Maldonado, Cerro de las Animas, 27.vii.1967 (*L*.*C*. *Zolessi and L*. *Benedetto*) (UDELAR).–Allotype, same data as holotype, except: 1 female.–Paratypes, same data, except: 3 males, 3 females, 8 nymphs.

#### Diagnosis

*Nymph*: metanotum with mid-distal margin (between wing pads) bilobated (W-shaped); femur and tibia lacking a fringe of bristles on the extensor margin, tarsi with setiform, small robust setae in the ventral face. *Adult*: male T10 in dorsal view provided with lateral sclerites separated from the anterior sclerite by a thin membrane; T10E short, with distal M-shaped border ending in two teeth. Male subgenital plate robust and with rounded distal margin. Male paraprocts with a ventral lobe sharply angulated, apex almost pointed; epiproct sclerotized absent. Female subgenital plate produced posteriorly in an M-shaped apex.

#### Redescription

*Paragripopteryx munoai* is a medium-sized species. ***Coloration*:** general color brown to dark brown. ***Head*:** dark brown with a lighter brown area between paired ocelli and coronal bifurcation, and two lighter brown bands from the lateral ocelli to the eyes; parietalia rough (Fig 11A and 11B). Three ocelli and compound eyes black. Antennae brown. Labrum brown. Maxillary palps brown, 5-segmented; first, second and fourth segments short, and third and fifth longer (Fig 11C). Labial palps brown, 3-segmented (Fig 11C). ***Thorax*:** dark brown; pronotum rough, square and with rounded corners. ***Legs*:** dark brown; tibia with a perpendicular suture in the proximal region and two spurs in the distal region. Tarsi brown, tarsomere 1 medium, tarsomere 2 short, and tarsomere 3 long. ***Wings*:** forewings slightly tinged with light brown spots, no pterostigmatic crossveins, RA unforked, RP and CuA forked (Fig 11D); hind wings with M3+4 fused to CuA, near their separation from M1+2, and separated before the wing margin (fork may be short or long); 6th anal vein can be fused to the wing margin (Fig 11D) although in some specimens it is not fused. ***Male abdomen*:** dark brown, membranous pleura between the abdominal segments 2–9. ***Male terminalia*:** in dorsal view, T10 dark brown, lateral sclerites separated from the anterior sclerite by a thin membrane; T10E short, with distal M-shaped border ending in two teeth (Fig 11E). In ventral view, subgenital plate dark brown, robust and with rounded apex (Fig 11F); paraprocts thin, directed to the T10E; epiproct sclerotized absent. In lateral view, paraprocts dark brown, with a ventral lobe sharply angulated, apex almost pointed (Fig 11G). Cerci multisegmented, cercomeres covered by thin bristles. ***Female abdomen*:** abdomen brown with a pair of lateral and anterior spots dark brown; segments 9–10 forming complete rings. ***Female terminalia*:** in dorsal view, T10 brown and with rounded apex. In ventral view, subgenital plate brown, produced posteriorly in a M-shaped apex covering the gonopore (Figs 8, 9 in [13]); paraprocts brown, covered with thin bristles and with rounded apex. ***Nymph*:** general color brown (yellowish in alcohol; Fig 11H); head brown, antennae with scape and pedicel brown, flagellum light brown (Fig 11I). Thorax brown and covered with spaced claviform bristles; pronotum rough with slightly rounded margins; wing pads brown, distal margin between wing pads bilobated (W-shaped) (Fig 11J). Femora thin, with some stout and short bristles. Tarsi in ventral face, with small robust setae (Fig 11K). Abdominal terga dark brown, segments 2–7 separated by membranous pleura; segments 8–10 forming complete rings; distal margin of T10 rounded with numerous stout and short bristles. Paraprocts robust, apex rounded (Fig 11L). Cerci brown. Gills light brown.

*Measurements*. Holotype, male: head width, 1.2 mm; pronotum width, 1.2 mm; pronotum length, 0.8 mm; forewing length, 6.75 mm; hind wing length, 6.0 mm; antennae length, 5.0 mm; paratypes, males (n = 3): head width, 1.1–1.2 mm; pronotum width, 1.1–1.3 mm; pronotum length, 0.7–0.8 mm; forewing length, 6.5–6.7 mm; hind wing length, 5.9–6.1 mm; antennae length, 5.0 mm (incomplete in some specimens, maximum length 6.0 mm); females (n = 4): head width, 1.4–1.6 mm; pronotum width, 1.4–1.5 mm; pronotum length, 1.0–1.1 mm; forewing length, 8.5–8.6 mm; hind wing length, 7.8–8.0 mm; antennae length, 6 mm; nymphs (n = 8): head width, 1.0–1.2 mm; pronotum width, 1.2–1.3 mm; pronotum length, 0.8–1.0 mm.

#### Comments

Based on the available material for study, nymphs of *P*. *munoai* can be distinguished from those of other *Paragripopteryx* species by the pronotum with the same head width, the metanotum with bilobated mid-distal margin, and the femora and tibiae extensor margins without a fringe of bristles. In addition, the nymphs of *P*. *munoai* are slenderer than those of other *Paragripopteryx*. Unlike other *Paragripopteryx* species, adults of *P*. *munoai* have no pterostigmatic crossveins in their forewings and the hind wings are half-elliptical and shortened from the angle of meeting of the CuP and AA1 veins to the distal margin of the wing. Our phylogenetic results indicate that this species is outside the clade that includes other species of *Paragripopteryx* (Clade C, Fig 2). Future studies may contribute to a better understanding of the positioning of this species among the South American Gripopterygidae genera.

#### Geographic distribution

The type locality of *P*. *munoai* is Cerro de las Animas in the Department of Maldonado, Uruguay [13]. The area is the second highest point in Uruguay, with an altitude of 501 m a.s.l. and a small summit area with steep slopes. This is the only locality where the species was recorded. For species geographic records, refer to the map in Fig 3.

### Paragripopteryx paranapiacabae Bispo and Lecci, 2011

(Fig 7I; Fig 9J–9L)

*Paragripopteryx paranapiacabae* Bispo and Lecci, 2011 [12]—Annales de Limnologie—International Journal of Limnology, 47: 379 (male, Figs 8–10).

*Material examined*. Holotype male, BRAZIL: São Paulo, Iporanga, Parque Estadual Intervales, Lago Negro, 19.xi.1992 (*C*.*G*. *Froehlich*) (MZSP).–Paratypes: same data as holotype, except: Ribeirão do Carmo (near Cachoeira das Pedrinhas), 01.xi.2002, 2 males (*A*.*S*. *Melo*).—***Other material***: BRAZIL: São Paulo, Iporanga, PETAR, 26–27.viii.2001, 1 male (*E*. *Galati*) (MZSP).

#### Diagnosis

*Adult*: general color ochraceous. Male forewings may have pterostigmatic crossveins incomplete or weak (Fig 7I). Male T10E elongate, base constricted, apex ending in two teeth (Fig 9J). Male paraprocts thick, L-shaped, apex rounded (Fig 9K); epiproct short, hook-shaped, without row of denticles on the inner margin (Fig 9L). Nymphs and females unknown.

#### Comments

*Paragripopteryx paranapiacabae* was described based on male specimens [12]. The material is in a good state of conservation. The elongate T10E and L-shaped paraprocts are striking for this species. A paratype specimen has paraprocts at an angle of 120° (Fig 9K). One male presented incomplete or weak pterostigmatic crossveins in its forewings.

#### Geographical distribution

The type locality of *P*. *paranapiacabae* is the Intervales State Park. However, the species was also recorded at the Alto Ribeira Tourist State Park (PETAR), both in São Paulo state, Brazil [12]. These two parks, together with the Carlos Botelho State Park, the Nascentes do Paranapanema State Park, the Xitué Ecological Station, the Serra do Mar (APA-SM) and the Quilombos do Meio Ribeira Environmental Protection Areas (APA-QMR), are part of the Paranapiacaba Continuum, an important ecological corridor of the Atlantic Forest in São Paulo state [58]. For the geographic records, refer to the map in Fig 3.

### Identification key

Below we provide a key to male adults of *Paragripopteryx*. The key does not include *P*. *baratinii*, a *nomen dubium*.

1. Epiproct upturned and sclerotized absent **2/2’**

1’. Epiproct upturned and sclerotized present (Fig 4C) **4/4’**

2. Forewings without pterostigmatic crossveins (Fig 11D). Male T10 without a conspicuous pair of unsclerotized indentation on the anterior margin. In lateral view, paraprocts with a ventral lobe sharply angulated, apex almost pointed (Fig 11G) ***Paragripopteryx munoai***

2’. Forewings with pterostigmatic crossveins (Fig 7A). Male T10 with a conspicuous pair of unsclerotized indentation on the anterior margin (Fig 6C; Fig 8J). In lateral view, paraprocts not as described above **3/3’**

3. T10E with apex bilobated, ending in two separated teeth (Fig 6C). Paraprocts progressively expanding along their length, apex concave-shaped (Fig 6D) ***Paragripopteryx ogum* sp. nov.**

3’. T10E with apex not bilobated, ending in two teeth close together (Fig 8J). Paraprocts progressively expanding to the middle and then narrowing, with a subapical tapering, apex rounded (Fig 8K) ***Paragripopteryx egena***

4. Paraprocts with distal region provided with black rod-like setae (Fig 8A, 8B; Fig 9D and 9E) **5/5’**

4’. Paraprocts with distal region not provided with black rod-like setae **6/6’**

5. T10 latero-distal margin with a conspicuous band not sclerotized on each side (but not open as clefts); T10E medium-length, spatulate-shaped and ending in two teeth separated by a small concave incision (Fig 8A) ***Paragripopteryx anga***

5’. T10 latero-distal margin with a conspicuous lateral cleft on each side; T10E elongate, Y-shaped and ending in two well-separated finger-shaped processes (Fig 9D) ***Paragripopteryx kapilei***

6. Epiproct short-length (Fig 4C; Fig 8M, 8N) **7/7’**

6’. Epiproct medium- to long-length (Fig 8B, 8C, 8P; Fig 9C; Fig 9H, 9I) **8/8’**

7. Subgenital plate rounded (Fig 2 in [16]). T10E forming a small process ending in two teeth close together (Fig 4B and 4E). Paraprocts with rounded tip (Fig 4C and 4F). Epiproct bending upward, almost falciform (Fig 4C) ***Paragripopteryx klapaleki***

7’. Subgenital plate trapezoidal (Fig 7 in [22]). T10E not forming a small process (half-elliptical), but also ending in two teeth close together (Fig 8L). Paraprocts with tip facing slightly backwards (Fig 8M). Epiproct shortened and finger-shaped (Fig 8M and 8N) ***Paragripopteryx guardae***

8. Epiproct finger-shaped, end rounded (Fig 9C; Fig 9H and 9I) **9/9’**

8’. Epiproct hook-shaped (tip facing inwards), end pointed (Fig 8E, 8I, 8P; Fig 9L) **10/10’**

9. T10 without an apparent extension, distal margin rounded with two small teeth close together and forming a tiny M-shaped projection (Fig 9A). Paraprocts without a subapical tooth (Fig 9B). Epiproct slender, sometimes exceeding the T10 and paraprocts, inner margin smooth (Fig 9C) ***Paragripopteryx intervalensis***

9’. T10 with an apparent extension, distal margin forming a median notch (M-shaped), ending in two separated teeth (Fig 9G). Paraprocts with a subapical tooth (Fig 9H). Epiproct robust, not exceeding the T10 and paraprocts, inner margin provided with two rows of tiny teeth (Fig 9I) ***Paragripopteryx merui***

10. T10 with a pair of conspicuous longitudinal parallel bands not sclerotized on the latero-distal margin (Fig 8O) ***Paragripopteryx hamata***

10’. T10 without sclerotized band on the latero-distal margin or, when present, not parallel **11/11’**

11. T10E with bilobated apex (dorsal view) (Fig 5C; Fig 9J) **12/12’**

11’. T10E without bilobated apex (dorsal view) (Fig 8D, 8G) **13/13’**

12. T10E medium-length, base wider than apex (not constricted), apex ending in an M-shaped incision (Fig 5C). Paraprocts not inclinated, tip triangular (Fig 5E) ***Paragripopteryx dasalmas* sp. nov.**

12’. T10E elongate and thin, base constricted, apex ending in a small concave incision (Fig 9J). Paraprocts strongly inclinated, apex rounded (Fig 9K) ***Paragripopteryx paranapiacabae***

13. T10E wide and rounded (Fig 8D). Paraprocts robust in their basal half, narrowing towards apex and ending in a sharp point (Fig 8E) ***Paragripopteryx blanda***

13’. T10E not wide and triangular-shaped (Fig 8G). Paraprocts with constant width along their lengths, apex rounded (Fig 8H) ***Paragripopteryx delicata***

## Discussion

### Phylogenetic relationships within *Paragripopteryx* of Clade C

Our data indicate that *Paragripopteryx* is not monophyletic, but that most species are grouped into a single clade (Clade C, Fig 2), which is only supported by a synapomorphy (presence of pterostigmatic crossveins in the forewings). According to our analyses, some internal relationships in *Paragripopteryx* (Clade C) still remain not well established (Fig 2). *Paragripopteryx intervalensis* was recovered as part of a polytomy nested in Clades D and E when we analyzed k = 3–5 (Fig 2). Likewise, *P*. *merui* was also included in this polytomy when k = 7–15 (S1 Fig). Despite the presence of two teeth at the end of the T10, *P*. *intervalensis* seems to have preserved a plesiomorphic shape of the T10 with a distal margin that does not protrude remarkably, a character shared with most *Gripopteryx* species. In this species, the paraprocts are devoid of complex shape and the epiproct is elongate, partially resembling that of *Aubertoperla illiesi*. Thus, it is likely that *P*. *intervalensis* is part of the first cladogenesis among the *Paragripopteryx* of Clade C. Another species, *P*. *merui*, was recovered either as part of Clade D (sister of Clade F, k = 3–5, Fig 2) or as part of a collapsed polytomy at the base of Clade C (k = 7–15, S1 Fig). Most characters that supported Clade D (characters 12 and 33) had a low CI and RI and a moderate to high homoplasy value, which may explain the fluctuation in the positioning of this species. Additionally, considering k = 3–5 (Fig 2), *P*. *hamata* and *P*. *paranapiacabae* were recovered as part of a polytomy with Clade G, while *P*. *ogum* and *P*. *dasalmas* were recovered as sister groups of Clades J and K, respectively. On the other hand, considering k = 7–15 (S1 Fig), these species were recovered as part of a polytomy with Clades H and K.

Some relationships remained unchanged independently of the weighting scheme. A relationship of particular interest is the one between *P*. *klapaleki*, *P*. *crassila*, and *P*. *guardae* (Clade F, Fig 2; Clade D, S1 Fig). Although these species have been recovered in a trichotomy supported by the falciform epiproct, it is possible to morphologically separate *P*. *klapaleki* and *P*. *crassila* from *P*. *guardae* according to the shape of the T10, paraprocts and subgenital plate in males, as well as the subgenital plate in females. In contrast, the wings, T10 and paraprocts of *P*. *klapaleki* are remarkably similar to those of *P*. *crassila*. As discussed in the section “Taxonomic Treatment”, the genitalia description of these two species are closely related to each other and the type of *P*. *crassila* did not show enough morphological differences from *P*. *klapaleki* to support its current status of distinct species. Thus, the synonymy of *P*. *crassila* with *P*. *klapaleki* was proposed in this study.

Other consistent clades were those formed by: 1) *P*. *blanda*, *P*. *delicata*, and *P*. *egena* (Clade H, Fig 2); and 2) *P*. *anga* and *P*. *kapilei* (Clade K, Fig 2), which were recovered in all analyses employed herein. The trichotomy nesting *P*. *blanda*, *P*. *delicata*, and *P*. *egena* was well supported by the trapezoidal shape of the T10 distal margin (11(1)) and 15(1) the presence of crossvein in the forewing RP fork, regardless of the k-values (Fig 2; S1 Fig). Despite the trichotomy, we found a closer morphological affinity between the T10 of males of *P*. *blanda* and *P*. *egena*, except for the conspicuous lateral sclerites in the latter. *P*. *blanda* and *P*. *egena* can be distinguished by the shape of the paraprocts in males, which are narrower and pointed in *P*. *blanda* and broadly rounded in *P*. *egena*, as well as by the presence of a sclerotized epiproct in the former (absent in *P*. *egena*). Lastly, our data revealed a close relationship between *P*. *anga* and *P*. *kapilei*. A prominent character that supported this relationship in all analyses was the paraprocts provided with a black rod-like setae in their distal region (34(1)), which presented maximum values for CI and RI. The epiproct and paraprocts were found to be remarkably similar in these two species, except for the most elongate paraprocts in *P*. *kapilei*. Moreover, while in *P*. *anga* the T10E is shorter and spatulate, in *P*. *kapilei* this structure is finger-shaped, with the T10 presenting a conspicuous lateral cleft on each side.

### Phylogenetic positioning and comments on *Paragripopteryx munoai*

Illies [18] erected *Jewettoperla* based on two species: *Gripopteryx crassila* [17] (proposed as the type species of *Jewettoperla*) and *Gripopteryx garbei* [59]. Later, Benedetto [13] described *Jewettoperla munoai* at the same time that Froehlich [16] considered *Jewettoperla* as a synonym of *Paragripopteryx*. As a result, *Jewettoperla munoai* was recognized as belonging to *Paragripopteryx* (*P*. *munoai*) [20]. However, our cladistic analysis places *P*. *munoai* outside of the *Paragripopteryx* delimitation (Clade C) and as sister of Clade B (Fig 2). Although the examination of the type series of *P*. *munoai* revealed characters in agreement with *Paragripopteryx*, such as the presence of the claviform bristles in nymphs, this character 1(2) was synapomorphic of a more inclusive clade containing *Aubertoperla illiesi* and *Gripopteryx cancellata* and probably lost in the ancestor of all other Gripopteryginae.

The differences between *P*. *munoai* and other *Paragripopteryx* species had already been noticed by Froehlich [22]. According to this author, *P*. *munoai* can be distinguished from its congeners by its eggs with a simple hemispherical shape, while the known pattern in species of Clade C (*P*. *klapaleki*, *P*. *anga*, and *P*. *blanda*) is elliptical in shape, provided by a differentiated chorion. In our analyses, two characters were responsible for differing nymphs of *P*. *munoai* from all other *Paragripopteryx*: the bilobated metanotum mid-distal margin (W-shaped, Fig 11J), whose shape in all other *Paragripopteryx* is straight, and the lack of a fringe of bristles on the extensor margin of the femora and tibiae in *P*. *munoai*, which is found in other *Paragripopteryx* species of Clade C.

*Paragripopteryx munoai* can also be distinguished from the species of Clade C by the details of the wings. Although Jewett [17] discussed the reliability of using crossveins as specific or generic characters of Gripopterygidae, our analyses showed the presence of pterostigmatic crossveins in the forewings as a character nesting most *Paragripopteryx* (Clade C), except for the forewings of *P*. *munoai* (Fig 11D). Despite being homoplastic at a broader level, this character presented a good informative sign indicated by a low homoplasy value and an RI slightly below 1. In the hind wings, an unusual character in Gripopteryginae (the sixth anal vein partially fused to the wing margin) was found in a few *P*. *munoai*–a condition also observed in *Aubertoperla illiesi* (Gripopteryginae), yet mostly commonly observed in genera of Antarctoperlinae, Dinotoperlinae, and Zelandoperlinae [23, 60]. Additionally, the wings in *P*. *munoai* are less slender than the patterns found in the *Paragripopteryx* species of Clade C.

Lastly, some considerations can be made about the nomenclature used by Benedetto [13] to describe the T10 in *P*. *munoai* as an “epiproct” (Figs 10–12 in [13]). As suggested by Froehlich [22] and verified in the type material, this species lacks an sclerotized epiproct (Fig 11E–11G). With regard to Benedetto’s scheme for *P*. *munoai*, the drawn structure is undoubtedly represented by a T10E (central sclerite) ending in two separated teeth. The T10 in this species is composed of anterior and central sclerites completely fused to each other and two lateral sclerites separated from the anterior sclerite by a thin membrane located distally to it.

### Final considerations

This study constitutes the first effort to understand more broadly the genus *Paragripopteryx*, including the relationships among its species under a cladistic approach. Herein, we analyzed a large number of specimens from different regions, and our data reinforced that the genus *Paragripopteryx* occurs mainly in streams from the Atlantic Rainforest. *Paragripopteryx* does not have a strong morphological differentiation from other genera and has a low variability among species, this configured a great challenge in our analyses. Indeed, although our study aimed to analyze the morphology of the genus *Paragripopteryx* in order to provide a revision of the genus based on its phylogeny, our results revealed that the proposition of a morphological phylogeny for *Paragripopteryx* is not a simple task, since most species of the genus (Clade C) are supported by only one plastic character, while one species (*P*. *munoai*) is retrieved outside the current boundaries of the genus.

To solve the polyphyletic issue of *Paragripopteryx*, a natural way would be to circumscribe the genus to species of Clade C and describe a new genus to allocate *P*. *munoai*. The problem is that Clade C is supported by a single synapomorphy: the presence of pterostigmatic crossveins in the forewings. In fact, the distinction of a new genus from *Paragripopteryx* (Clade C) would be mainly based on the absence of pterostigmatic crossveins in the forewings; however, we observed that the veins can be variable, revealing the fragility of this character. In addition, some details of the nymphal morphology could also be used; however, the small variation in these characters could be compared to the interspecific variation within *Paragripopteryx*. In this scenario, before taking the decision to describe a new genus, it is important to expand the sampling effort in Uruguay in an attempt to obtain new specimens of *P*. *munoai* and locate *P*. *baratinii* in its type locality. These specimens could be the starting point of a broader phylogenetic analysis based on a more integrative approach (including both morphological and molecular data), allowing the proposal of a new genus of Gripopterygidae on more solid bases. Nonetheless, although we take a more conservative taxonomic position, we indicate that *Paragripopteryx* is probably restricted to Clade C species.

In addition to the phylogenetic analyses, in this study we described nymphs and new species of *Paragripopteryx*, made taxonomic adjustments, and updated the distribution of several species. In this context, some of the important taxonomic contributions to the knowledge of the genus were: 1) the descriptions of an advanced nymphal exuviae of *P*. *intervalensis* and the nymph of *P*. *kapilei*; 2) the descriptions of two new species, *P*. *dasalmas* and *P*. *ogum*; 3) the synonymization of *P*. *crassila*, which was considered *P*. *klapaleki*; and 4) the consideration of *P*. *baratinii* as *nomen dubium*. Therefore, it was concluded that *Paragripopteryx* has 14 species (13 belonging to Clade C), nine of which have nymphs described (eight belonging to Clade C) (Table 2). It is worth mentioning that the present study is one of the first revisions of a South American Gripopterygidae genus using a phylogenetic approach and that most South American Gripopterygidae genera still need revision [23, 24]. Lastly, this study also aimed to add important information to better understand the New World genera of this family and create foundations for the next steps.

**Table 2:** Summary of the current taxonomic treatments of *Paragripopteryx* and their respective records and described stages (new geographic records in bold).

No.	Species	Author, year	Geographic records	Known life stages		
				♂	♀	Ny
*Paragripopteryx*						
1	*P*. *anga*	Froehlich, 1969 [16]	BRA (SP)	●	●	●
2	*P*. *blanda*	Froehlich, 1969 [16]	BRA (SP, **SC**)	●	●	●
3	*P*. *delicata*	Froehlich, 1994 [22]	BRA (SP)	●	●	●*
4	*P*. *egena*	Froehlich, 1994 [22]	BRA (ES, SP, **SC**)	●	●	-
5	*P*. *guardae*	Froehlich, 1994 [22]	BRA (SP)	●	●	●
6	*P*. *hamata*	Froehlich, 1994 [22]	BRA (SP)	●	●	-
7	*P*. *intervalensis* ^**+**^	Bispo and Lecci, 2011 [12]	BRA (ES, **MG**, **SC**, SP)	●	●	●*
8	*P*. *kapilei* ^**+**^	Bispo and Lecci, 2011 [12]	BRA (SP, **SC**)	●	-	●
9	*P*. *klapaleki*	Enderlein, 1909 [7]	ARG (MIS); BRA (RJ, **SC**, SP)	●	●	●
10	*P*. *merui*	Froehlich, 1994 [22]	BRA (SP, **ES**)	●	-	●*
11	*P*. *munoai*	Benedetto, 1969 [13]	URY (MAL)	●	●	●
12	*P*. *paranapiacabae*	Bispo and Lecci, 2011 [12]	BRA (SP)	●	-	-
New species described in this study						
13	*P*. *dasalmas* sp. nov.	In this paper	BRA (**BA**)	●	●	-
14	*P*. *ogum* sp. nov.	In this paper	BRA (**SC**)	●	●	-
Synonymy						
	*P*. *crassila* ^**S**^	(Jewett, 1960) [17]	BRA (SC)	●	●	-
*Nomen dubium*						
	*P*. *baratinii* ^**L**^	Benedetto, 1983 [14]	URY (LAV)	●	●	-

***Abbreviations***: ARG–Argentina; BA–Bahia state; BRA–Brazil; ES–Espírito Santo state; LAV–Department of Lavalleja; MAL–Department of Maldonado; MG–Minas Gerais state; MIS–Province of Misiones; RJ–Rio de Janeiro state; SC–Santa Catarina state; SP–São Paulo state; URY–Uruguay. ***Symbols***: ♂–male; ♀–female; Ny–nymph; +–nymphs or exuviae described in the current study

*–description based on last instar exuviae; L–lost material; S–synonym of *P*. *klapaleki*. ***Note***: In addition to species records, immature specimens of the genus were also recorded in Rio Grande do Sul state, Brazil without specific identification [48].

## Supporting information

S1 Table Character matrix. Characters and character states of *Paragripopteryx* and outgroup species.
(PDF)

S1 Fig Analysis under IW scheme (k = 7–15). Consensus strict from two trees; most *Paragripopteryx* (written in blue) nested in a clade, except for *P*. *munoai* (written in red).
(TIF)
