# Supplementary material for: Systematic revision and phylogeny of Paragripopteryx Enderlein, 1909 (Plecoptera: Gripopterygidae)
Source: PLoS One. 2022 Mar 3;17(3):e0264264. doi: 10.1371/journal.pone.0264264 (PMC8893681; doi:10.1371/journal.pone.0264264)
Supplement: S1 Table — Characters and character states of Paragripopteryx and outgroup species. (PDF) [file pone.0264264.s001.pdf]

S1 Table.

Taxons	Character states			
	0000000001<br>1234567890	1111111112<br>1234567890	2222222223<br>1234567890	33333333<br>12345678
Ceratoperla fazi	01001000-1	0001000000	1021100-11	51000300
Alfonsoperla flinti	01010010-0	2201010000	?0120-0-11	00001??4
Riekoperla karki	000111?1?0	0000-01101	11210?0-11	410013??
Aubertoperla illiesi	2004111120	0001010001	1021100-01	411004??
Claudioperla tigrina	0004111100	2201010101	0020101101	31000305
Limnoperla jaffueli	0002112100	0200-11101	0100101001	51001314
Potamoperla myrmidon	0004112100	0101010101	0020111100	--000314
Rhithroperla rossi	0004112120	0101010101	0122101101	30000211
Teutoperla rothi	0004112100	2?00010101	0000000-01	3100131?
Uncicauda testacea	0002002100	2001010?01	?000101000	--000414
Tupiperla gracilis	0000002100	2001010111	0000111100	--000005
Tupiperla robusta	0000002100	2001010111	0000101100	--001004
Guaranyperla guapiara	1010102110	2011010111	0000101100	--000314
Guaranyperla nitens	1010102110	3011010111	0000111100	--000313
Gripopteryx cancellata	2103111121	1001110101	01000-0-01	31000315
Paragripopteryx anga	2004112120	0011110101	0000111101	10110014
Paragripopteryx blanda	2004112120	1011110101	0000101101	10001005
Paragripopteryx crassila	??????????	??11010101	0000101101	2?100315
Paragripopteryx dasalmas sp. nov.	??????????	??11010101	0000111101	1000?014
Paragripopteryx delicata	2004112120	1211111101	0000101101	10000015
Paragripopteryx egena	??????????	??11010101	0020101100	--100015
Paragripopteryx guardae	2004112120	0211010101	0000101101	21100114
Paragripopteryx hamata	??????????	??11010101	0000101101	10000311
Paragripopteryx intervalensis	2004112120	0011010101	00000-1101	41000315
Paragripopteryx kapilei	2004112120	0211010101	0100111101	101100??
Paragripopteryx klapaleki	2004112120	0211010101	0000101101	21100315
Paragripopteryx merui	2004112120	0211010101	0000111101	411000??
Paragripopteryx munoai	2005001120	0001010*01	0000111100	--001302
Paragripopteryx ogum sp. nov.	??????????	??11010101	0000111100	--0000?5
Paragripopteryx paranapiacabae	??????????	??11010101	0100101101	100003??
