# Supplementary figures and images for: Systematic revision and phylogeny of Paragripopteryx Enderlein, 1909 (Plecoptera: Gripopterygidae)
Source: PLoS One. 2022 Mar 3;17(3):e0264264. doi: 10.1371/journal.pone.0264264 (PMC8893681; doi:10.1371/journal.pone.0264264)

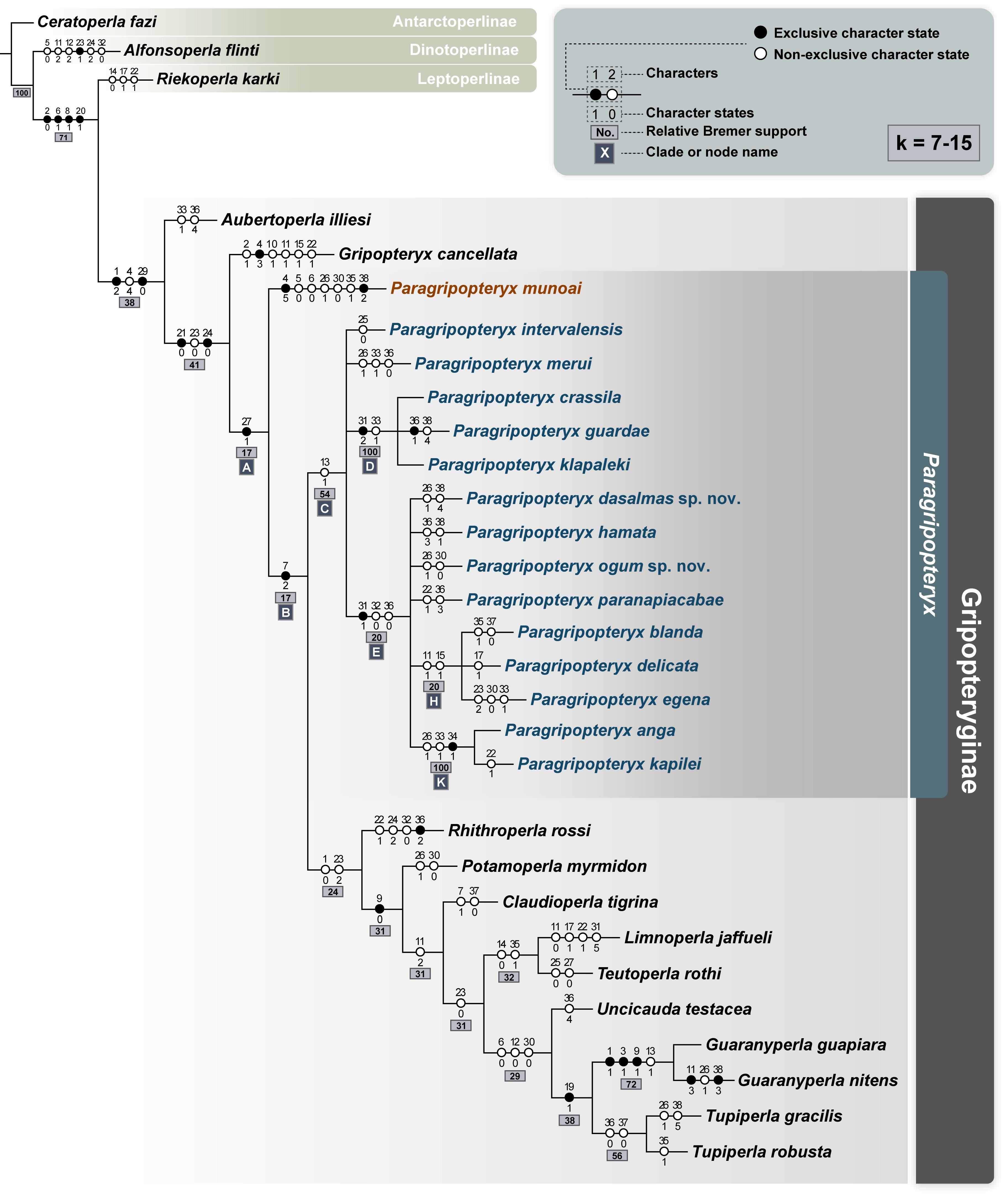

Supplement: S1 Fig — Consensus strict from two trees; most Paragripopteryx (written in blue) nested in a clade, except for P. munoai (written in red). (TIF) [file pone.0264264.s002.tif]
